# The WD40-domain containing protein CORO2B is specifically enriched in glomerular podocytes and regulates the ventral actin cytoskeleton

**DOI:** 10.1038/s41598-017-15844-1

**Published:** 2017-11-21

**Authors:** M. Rogg, M. Yasuda-Yamahara, A. Abed, P. Dinse, M. Helmstädter, A. C. Conzelmann, J. Frimmel, D. Sellung, M. L. Biniossek, O. Kretz, F. Grahammer, O. Schilling, T. B. Huber, C. Schell

**Affiliations:** 1 Department of Medicine IV, Medical Center – University of Freiburg, Faculty of Medicine, University of Freiburg, Freiburg, Germany; 2 Department of Medicine, Shiga University of Medical Science, Otsu, Shiga, Japan; 3 Institute of Molecular Medicine and Cell Research, University of Freiburg, Freiburg, Germany; 4 Institute of Anatomy and Cell Biology, Dept. for Neuroanatomy, Medical Faculty, Albert-Ludwigs-University Freiburg, Freiburg, Germany; 5 III. Department of Medicine, University Medical Center Hamburg-Eppendorf, Hamburg, Germany; 6 BIOSS Center for Biological Signalling Studies and Center for Systems Biology (ZBSA), Albert-Ludwigs-University, Freiburg, Germany; 7 German Cancer Consortium (DKTK) and German Cancer Research Center (DKFZ), Heidelberg, Germany; 8 Institute for Surgical Pathology, Medical Center Freiburg, Freiburg, Germany; 9 Berta-Ottenstein Programme, Faculty of Medicine, University of Freiburg, Freiburg, Germany

## Abstract

Podocytes are highly specialized epithelial cells essentially required to establish and maintain the kidney filtration barrier. Due to their complex cellular architecture these cells rely on an elaborated cytoskeletal apparatus providing plasticity as well as adaptive adhesion properties to withstand significant physical filtration forces. However, our knowledge about podocyte specific components of the cytoskeletal machinery is still incomplete. Employing cross-analysis of various quantitative omics-data sets we identify the WD40-domain containing protein CORO2B as a podocyte enriched protein. Furthermore, we demonstrate the distinct localization pattern of CORO2B to the ventral actin cytoskeleton serving as a physical linkage module to cell-matrix adhesion sites. Analysis of a novel *Coro2b* knockout mouse revealed that CORO2B modulates stress response of podocytes in an experimental nephropathy model. Using quantitative focal adhesome proteomics we identify the recruitment of CFL1 via CORO2B to focal adhesions as an underlying mechanism. Thus, we describe CORO2B as a novel podocyte enriched protein influencing cytoskeletal plasticity and stress adaptation.

## Introduction

Glomerular epithelial cells (namely podocytes) represent together with endothelial cells and the glomerular basement membrane (GBM) essential components of the kidney filtration barrier^1,2^. Podocytes enclose glomerular capillaries with a network of interconnected cellular protrusions, which are structurally divided into primary and secondary processes^1^. Podocytes require an efficient adhesion to the GBM in order to withstand constant exposure to physical forces and prevent detachment into the urinary space^3^. This elaborate adhesion machinery consists of a multiprotein complex also known as the focal adhesome^3–5^.

Increased permeability of the kidney filtration barrier, causing loss of plasma proteins to the urine (proteinuria), is one major symptom of progressive glomerular and chronic kidney disease. An uniform pattern of any podocyte disease is the progressive retraction of the foot process network, commonly termed as foot process effacement (FPE -^6^). Detachment of podocytes from the GBM into the urinary space is a major contributing factor for kidney disease progression^3,7,8^. The identification of disease causing mutations within actin cytoskeleton associated genes or focal adhesion complex components underlines the importance of both structures for podocyte function (e.g. mutations in ACTN4 and ITGA3 –^9–13^). However, it is still poorly understood, how and if podocyte specific molecules contribute to the maintenance of either the cytoskeleton or FAs. However, this knowledge represents a prerequisite for the development of novel podocyte specific diagnostic approaches or therapies for glomerular disease.

The coronin family of actin regulators is well known to control actin dynamics and turnover^14^. One unifying motive of this protein family is an unique WD40 domain^15^. Coronins are grouped into 3 types based on phylogenetic analysis^16^. Type 1 coronins were extensively characterized, since mutations within *Coro1a* are associated with severe combined immune deficiency syndromes (SCID) in humans^16–18^. Functionally, balancing of Arp2/3 based actin assembling and ADF/cofilin based actin filament disassembly was linked to type 1 coronins^19,20^. On the contrary, type 2 coronins are much less studied, but seem to recruit stronger to actin fibers and focal adhesions^21,22^. While *Coro2a* was linked to the regulation of focal adhesion turnover a comprehensive functional description of *Coro2b* is still missing^21^.

Here, we re-analyzed transcriptomic and proteomic datasets for the coronin family of actin regulators and thereby identified CORO2B as a novel highly podocyte specific expressed protein^5,23^. By combining different labeling modalities like *mRNA in situ* hybridization, LacZ reporter mice, immunogold EM and live cell imaging we could comprehensively describe the *in vivo* expression and subcellular localization of *Coro2b*. Generation of a novel constitutive *Coro2b* knockout mouse model revealed a protective effect for *Coro2b* in situations of experimental podocyte stress. Finally, employing quantitative focal adhesion proteomics identified CORO2B as a modulator of CFL1 recruitment to the ventral filamentous actin/focal adhesion interface.

## Results

### *Coro2b* is highly expressed in developing and mature podocytes

**Figure 1:**
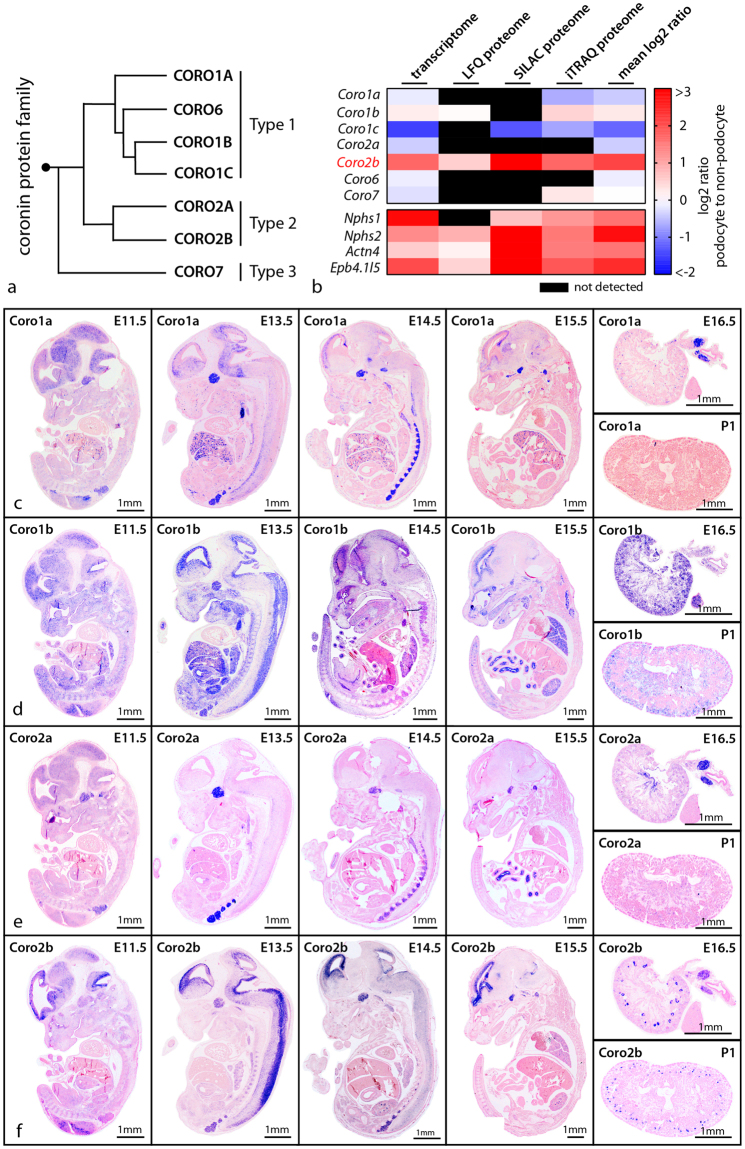
*Coro2b* is a novel podocyte specific expressed gene. (**a**) A phylogenetic tree of the coronin family of WD40-domain containing proteins was generated by comparison of *coronin* sequences using Clustal Omega. (**b**) Re-analysis of published *omics* datasets comparing protein expression of podocytes to glomerular non-podocyte cells identifies *Coro2b* as a novel podocyte specific expressed protein (previously described podocyte specific proteins are depicted as reference). (**c–f**) mRNA *In-Situ*-Hybridization of coronin family members *Coro1a*, *Coro1b*, *Coro2a* and *Coro2b* confirmed a distinct expression of *Coro2b* in glomeruli of the developing kidney. Furthermore, *Coro2b* expression was detected in the developing nervous system as well as developing pituitary gland.

**Figure 2:**
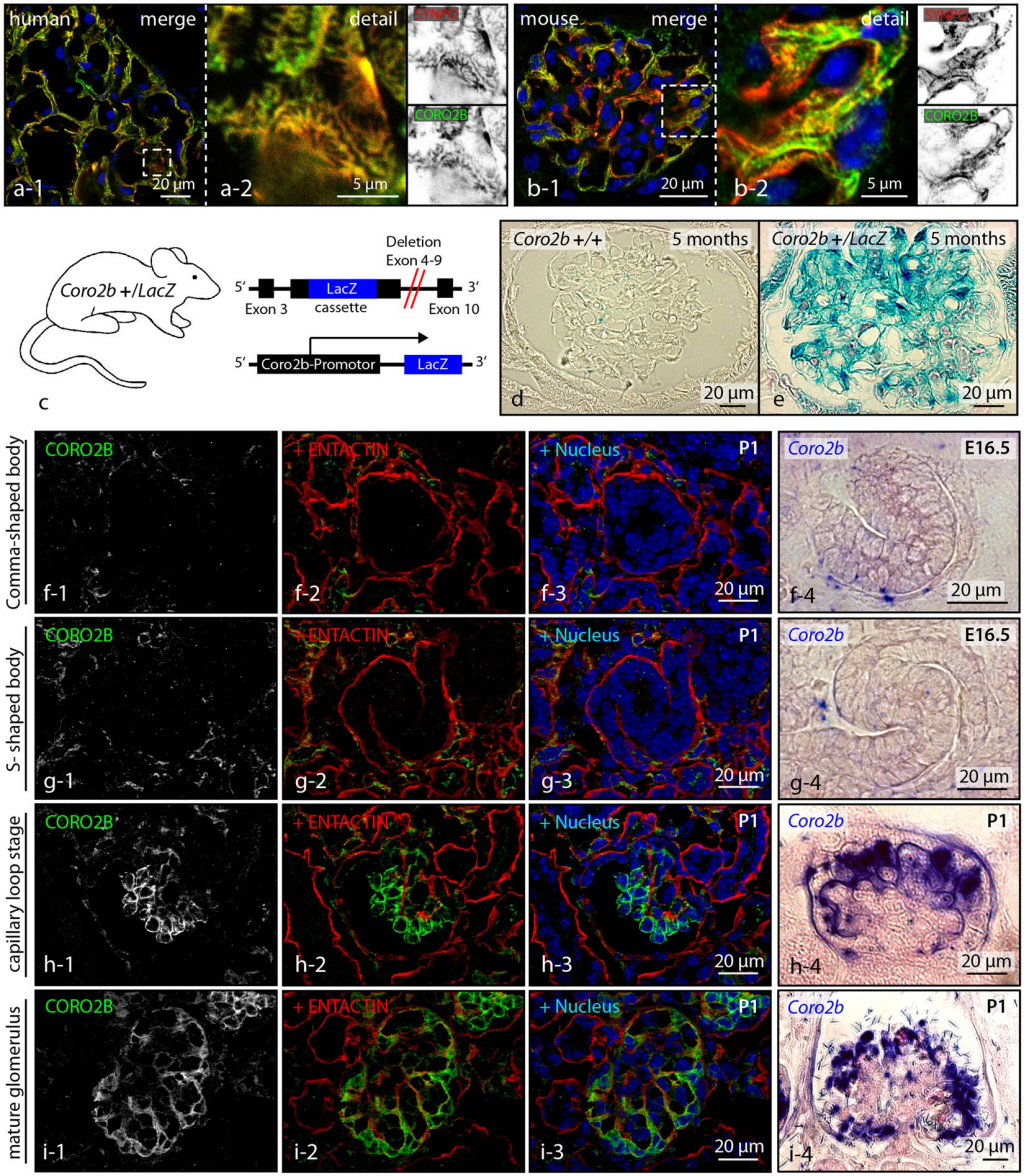
*Coro2b* is highly expressed in developing and adult podocytes. (**a,b**) Immunofluorescence staining for CORO2B and SYNPO could detect a strong expression of CORO2B in podocytes of adult mouse and human glomeruli. (**c–e**) A *Coro2b* promotor driven *LacZ-*reporter allele shows strong *Coro2b* promotor activity in glomeruli of adult mice. (**f–i**) *Coro2b* expression during kidney development was detected earliest at the capillary loop stage, where capillary and mesangial cells start to intrude into the podocyte compartment; formation of primitive foot processes starts to occur at the same developmental stage (ENTACTIN was used to label the glomerular basement membrane). *In situ*-hybridization for *Coro2b mRNA* detected respective expression patterns.

To discover novel podocyte specific regulators of the actin cytoskeleton, transcriptome and proteome datasets of isolated podocytes were re-analyzed for the expression of the coronin family of actin cytoskeleton regulators (Fig. 1a,b). Here, CORO2B was identified due to a high enrichment within the podocyte compartment, indicative of a potentially podocyte-specific protein. Based on this screen *Coro1a*, *Coro1b*, *Coro2a* and *Coro2b* were chosen for further validation by mRNA *in-situ* hybridization (Fig. 1c–f). Here, a pronounced *Coro2b* expression was observed in glomeruli of developing kidneys (E 14.5 on) as well as glomeruli of newborn mice. Additionally, a strong *Coro2b* expression in the developing nervous system and developing pituitary gland was detected (Fig. 1f). Interestingly, also a strong expression of *Coro1b* was detected in the whole nephrogenic zone of developing kidneys, but overall at much lower levels in kidneys of newborn mice (Fig. 1d). *Coro1a* and *Coro2a* expression was not detected in the glomerular compartment (Fig. 1c,e). Based on these findings *Coro2b* was selected for a more detailed validation and description of gene expression. On protein levels CORO2B was strongly detected in podocytes of adult human and mice kidneys (Figs 1a and 2a,b). These observations were corroborated by the generation and analysis of a *LacZ* reporter mouse (reflecting *Coro2b* promotor activity - Fig. 2c). In podocytes of developing glomeruli *Coro2b* expression was first detected at the capillary loop stage (Fig. 2f–i).

### Foot processes, ventral actin cytoskeleton and focal adhesions represent subcellular localization sites of CORO2B

**Figure 3:**
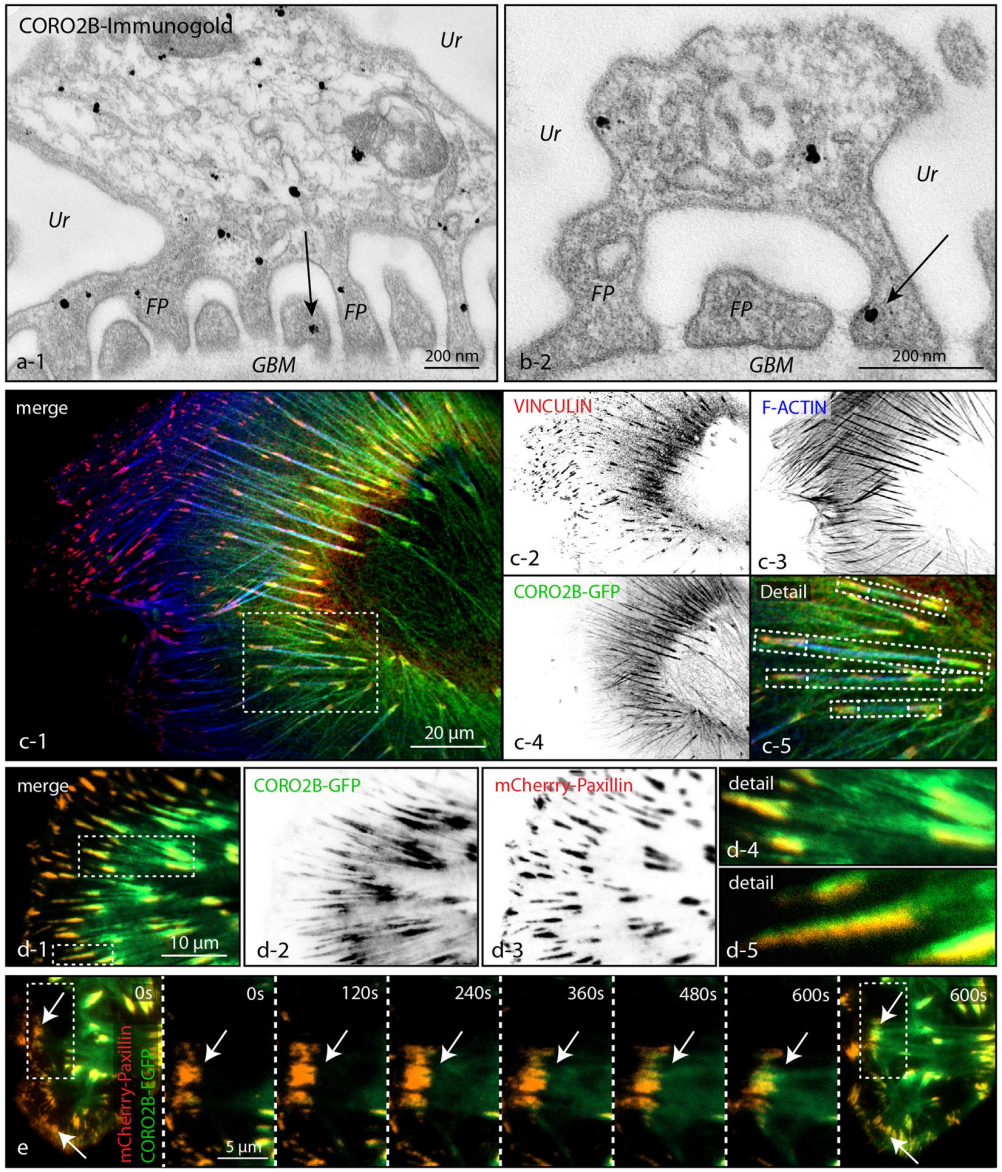
CORO2B localizes to foot processes, the ventral actin cytoskeleton and focal adhesions. (**a,b**) CORO2B immunogold labeling of glomerular TEM sections shows a prominent localization to the podocyte cell corpus, primary and secondary processes. (**c**) Immunofluorescence staining for F-ACTIN, VINCULIN and CORO2B-GFP in human immortalized podocytes reveals localization of CORO2B to ventral actin stress fibers and ventral focal adhesions. (**d,e**) TIRF life cell imaging for CORO2B-GFP and mCherry-PAXILLIN in human immortalized podocytes confirms pronounced localization of CORO2B to the ventral stress fiber - focal adhesions complex and shows focal adhesion recruitment of CORO2B at the late focal adhesion maturation phase.

Employing immunogold labeling and electron microscopy revealed a wide distribution pattern of CORO2B to the podocyte cell corpus as well as foot processes (Fig. 3a). Immortalized human podocytes were used for detailed analysis of the subcellular localization of CORO2B. Here CORO2B was detected at actin stress fibers and focal adhesion sites (Fig. 3 c–e, and supplemental Fig. 1). Interestingly, CORO2B predominantly localized to the ventral actin cytoskeleton, which is defined by the presence of fibrillary focal adhesions connected to central stress fibers (Fig. 3c,d)^24^. Live cell TIRF imaging demonstrated a pronounced recruitment for CORO2B towards mature focal adhesions. Here, we observed a tendency for CORO2B to localize at the proximal side of focal adhesions, representing the linkage site with the actin cytoskeleton (Fig. 3e). CORO2B recruitment to the F-actin/focal adhesion interface was furthermore supported by association of CORO2B with F-actin binding focal adhesion proteins like VINCULIN and TALIN, but not with focal adhesion proteins without actin binding properties like PAXILLIN as demonstrated by co-immunoprecipitation experiments (supplemental Fig. 1).

### Constitutive knockout of *Coro2b* does not impair mice survival and glomerular function, but influences podocyte stress response

**Figure 4:**
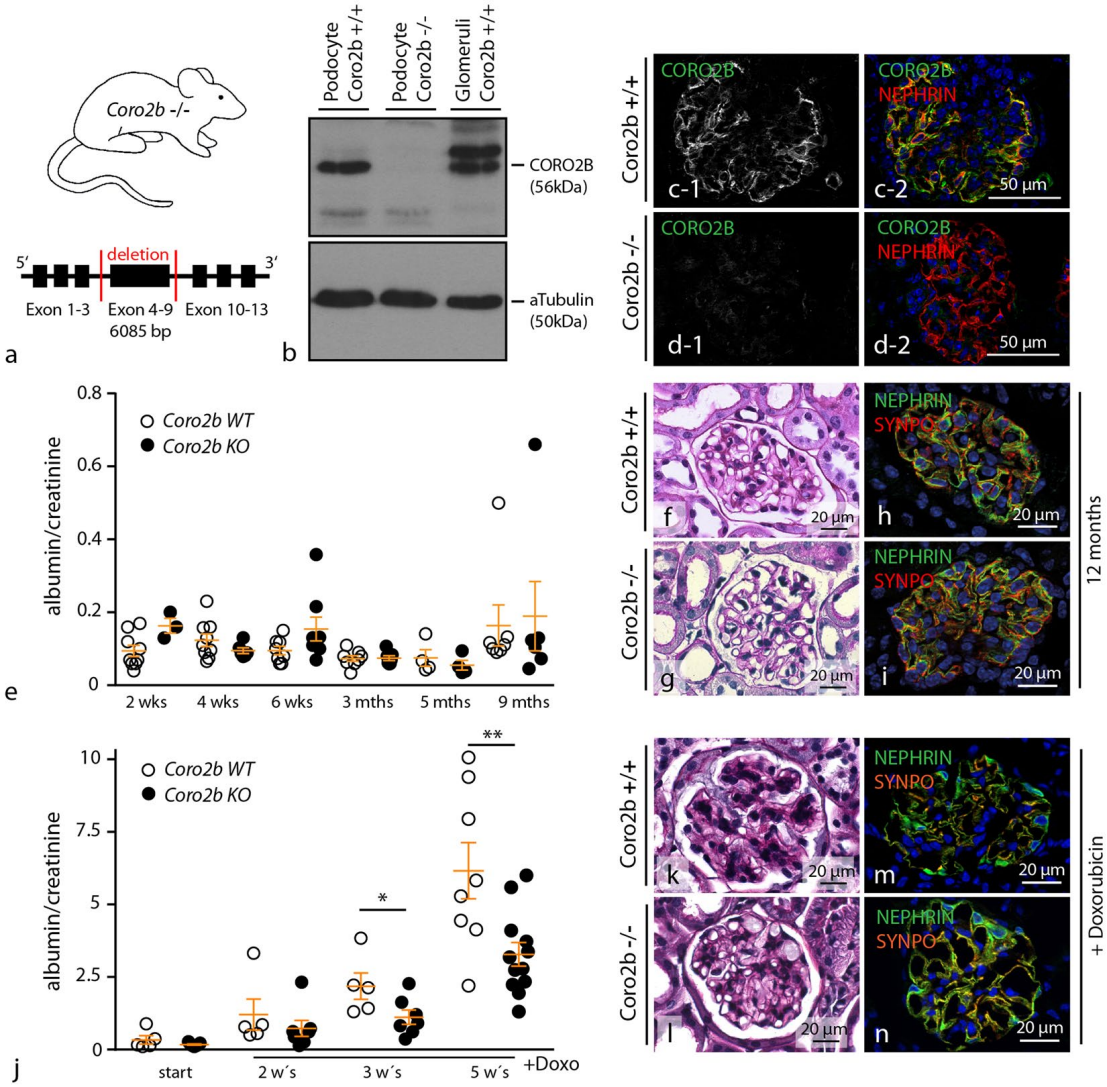
Loss of CORO2B does not impair glomerular function, but influences stress response to Doxorubicin treatment. (**a**) Schematic illustrating strategy for *Coro2b* knockout generation by deletion of Exon 4–9. (**b,c**) *Coro2b* knockout was confirmed by western blotting of isolated podocytes and immunofluorescence staining of kidney cryo-sections. The slit-diaphragm component NEPHRIN was used to co-label the podocyte compartment. (**e**) Albumin to creatinine ratio (ACR) indicates normal glomerular function in *Coro2b* knockout animals up to 9 months. (Individual animals were indicated as dots.) (**f–i**) 12 months old *Coro2b* knockout animals showing normal glomerular histology in PAS staining as well as normal expression and localization of the slit diaphragm component NEPHRIN and the podocyte specific actin cytoskeleton protein SYNPO. (**j**) *Coro2b* knockout mice exhibit increased resistance to Doxorubicin induced podocyte damage, indicated by higher ACR in wild type animals after 3 and 5 weeks of treatment. (5 *WT* and 7 *KO* animals were analyzed at indicated time points, for measurement after 5 weeks of treatment an additional experimental run of 3 *WT* and 5 *KO* animals was included; *p < 0.05, **p < 0.01; for detailed information see statistical section) (**k–n**) PAS staining and immunofluorescence staining for NEPHRIN and SYNPO of Doxorubicin treated animals reveals modest mesangial expansion and decreased levels of bona fide podocyte markers in wild type mice.

**Figure 5:**
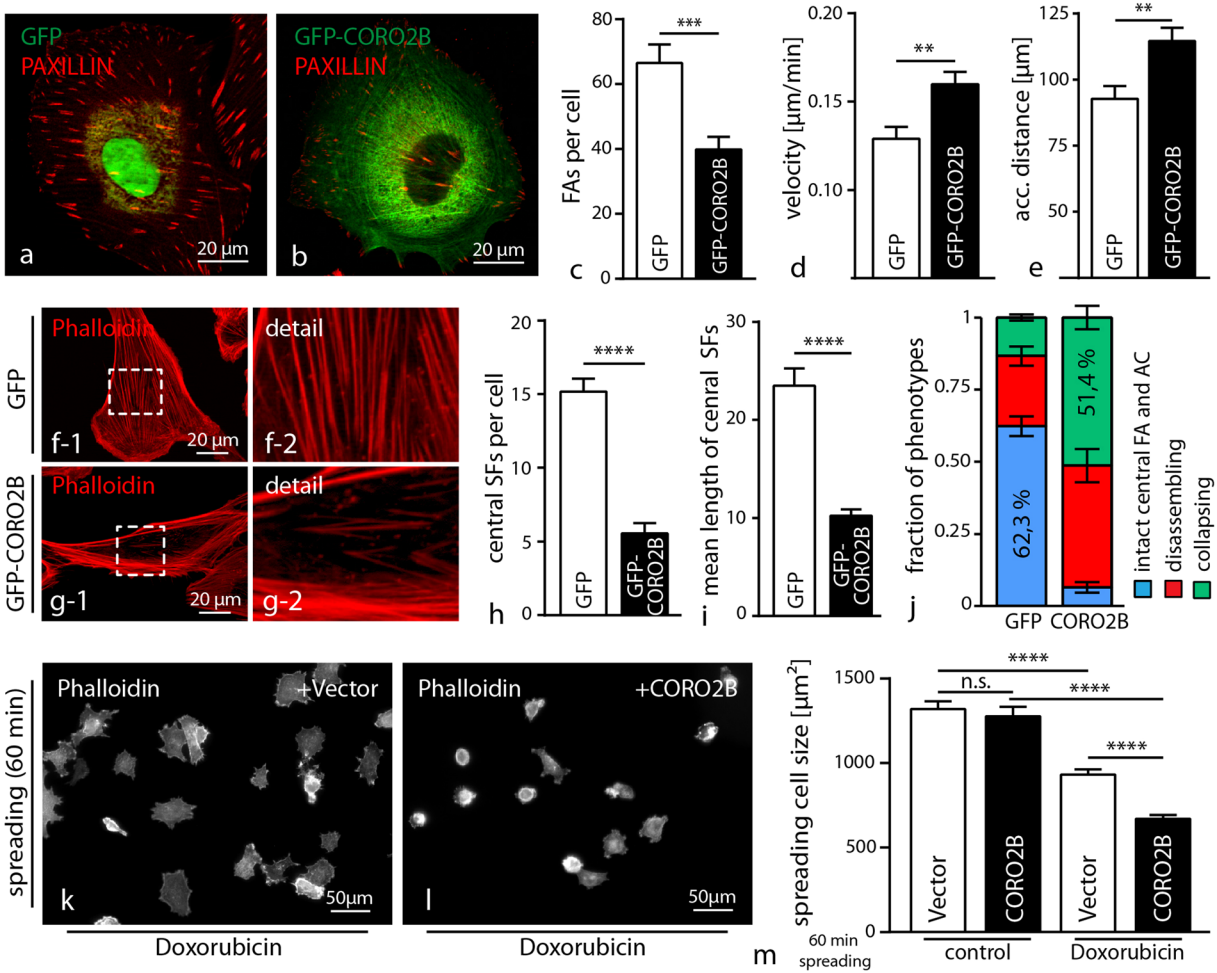
CORO2B in human podocytes augments susceptibility to cellular stress. (**a–c**) Expression of CORO2B in human podocytes leads to a decrease in mean focal adhesion number per cell. Focal adhesions were visualized by PAXILLIN immunofluorescence staining. (n = 12 control and 12 CORO2B cells of one representative experiment were analyzed; >3 independent experiments were performed; ***p > 0.001), (**d–e**) Increased CORO2B expression enhances migratory capability of podocytes in single cell migration assays (n = 117 control and 100 CORO2B cells out of 3 independent experiments; **p < 0.01). (**f–j**) CORO2B expression results in a decrease of central actin cytoskeleton structures. F-ACTIN was visualized by Phalloidin staining. (n = 30 control and 36 CORO2B cells of one representative experiment were analyzed for central stress fibers (SF), >3 independent experiments were performed; ****p > 0.0001; cell type fraction was analyzed in 3 independent experiments by phenotyping of 100 cells per condition and experiment, data are significant (p < 0.001) for difference in intact central FA and AC group.) (**k–m**) CORO2B increases susceptibility to Doxorubicin induced cellular spreading defects. Cell spreading was performed on collagen IV coated glass cover slips for 1 hour. Podocytes were continuously treated with 1 µg/ml Doxorubicin for 24 hours before and during cell spreading assay (n = 329 V. ctrl., 327 CORO ctrl., 356 V. Doxo., 312 CORO. Doxo., cells of 3 independent experiments were analyzed per condition; ****P > 0.0001).

To test the *in vivo* relevance of *Coro2b* for podocyte function we generated a novel constitutive *Coro2b* knockout model on a C57/BL6 genetic background (Fig. 4a). Western blot experiments of isolated podocyte lysates and immunofluorescence staining confirmed CORO2B expression in wild-type animals and conclusively absent expression in podocytes of *Coro2b* deficient mice (Fig. 4b–d and supplemental Fig. 2). Constitutive *Coro2b* knockout animals were born alive in normal Mendelian distributions and no obvious phenotype was observed at age of birth. Female and male *Coro2b* knockout animals were fully fertile and showed normal litter size. Compared to *WT* no obvious phenotype or difference in survival was observed up to an age of 18 months. Glomerular function of *Coro2b* knockout mice was analyzed by measuring albumin to creatinine ratio in spot urine samples and revealed a normal function of the kidney filtration barrier in *KO* animals (Fig. 4e). These physiological parameters were reflected by a rather unaffected glomerular histology as well as unaltered expression and localization of slit-diaphragm and cytoskeleton proteins in respective *KO* animals (Fig. 4f–i and supplemental Fig. 3). We therefore assumed that absence of CORO2B does not lead to any obvious phenotype or detectable impairment of the kidney filtration barrier. To test for a possible role of *Coro2b* in glomerular stress response we exposed control and *Coro2b* deficient animals to the established Doxorubicin-nephropathy model. Treatment by the anthracycline Doxorubicin is an established model to induce a FSGS like phenotype in mice^25^. Interestingly, *Coro2b* knockout animals were partially protected as they developed significantly lower levels of proteinuria after 3 and 5 weeks of treatment (Fig. 4j). On a histological level, PAS staining and immunofluorescence staining for NEPHRIN and SYNPO revealed pronounced features of damage in wild type animals when compared to respective knockout animals (Fig. 5k–n – of note, CORO2B expression and localization was not impaired in Doxorubicin treated *WT* animals; supplemental Fig. 4). We therefore concluded that the absence of CORO2B does partially protect glomerular function and the glomerular filtration barrier of Doxorubicin treated animals.

### CORO2B controls the ventral F-actin/focal adhesion interface and augments susceptibility to cellular stress

**Figure 6:**
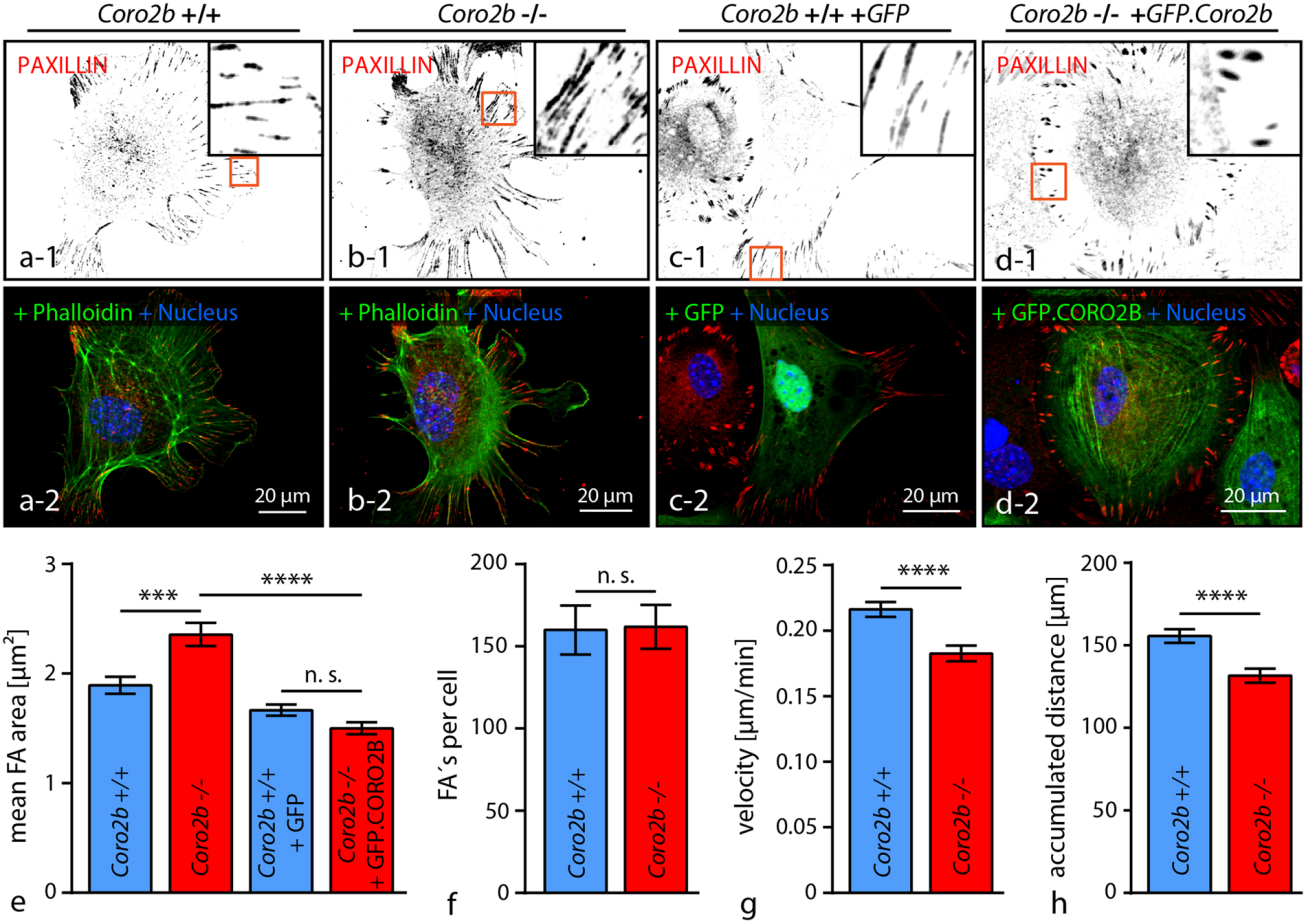
*Coro2b* knockout influence focal adhesion dynamics. (**a–f**) Isolated MEF from *Coro2b* knockout and wild type mice show increased average focal adhesion size but normal focal adhesion numbers in *KO* cells. Re-expression of CORO2B rescued average focal adhesion size compared to control cells. Immunofluorescence staining visualizes F-ACTIN (Phalloidin) or GFP and the focal adhesion component PAXILLIN (n = 40 *WT*, 43 *KO*, 33 *WT* + GFP, 31 *KO* + CORO cells out of 3 independent experiments were analyzed; n.s. – non significant, ***p < 0.001, ****p < 0.0001; one way ANOVA and Tukey’s multiple comparisons test was applied). (**g–h**) Knockout of *Coro2b* reduces migratory capability of podocytes in single cell migration assays (n = 252 *WT* and 237 *KO* cells out of 3 independent experiments were analyzed; ****p < 0.0001).

Cultured immortalized human podocytes express *Coro2b* on only lowest detectable levels. Therefore re-expression of *Coro2b* in human podocytes was performed to further analyze the impact of *Coro2b* expression on the actin cytoskeleton and podocyte stress responses (Fig. 5a,b and supplemental Fig. 4). Interestingly, only re-expression of *Coro2b* leads to a reduced number of focal adhesions per cell in human immortalized podocytes (Fig. 5a–c). Conclusively, a reduced cell-substratum adhesion was reflected by enhanced migratory capability in single cell migration assays (Fig. 5d,e). Detailed analysis of the actin cytoskeleton architecture revealed a pronounced loss of the central/ventral actin cytoskeleton in *Coro2b* expressing cells (Fig. 4f–j). To finally test the functional relevance of *Coro2b* on Doxorubicin induced podocyte stress, control and *Coro2b* re-expressing podocytes were compared in spreading assays. Here, *Coro2b* re-expressing and control podocytes were pre-treated with Doxorubicin and spreading assays were performed on collagen IV coated glass cover slips for 60 minutes. Remarkably, Doxorubicin treated *Coro2b* re-expressing podocytes showed a decreased spreading capability and disarranged actin cytoskeleton formation compared to treated control cells (Fig. 4k–m). As also control cells showed impaired spreading under treatment conditions, our findings suggest that CORO2B might serve as a susceptibility factor for a general stress response phenotype in cultured podocytes (Fig. 5m). In general, the ventral actin cytoskeleton/focal adhesion interface exhibits a high level of susceptibility in podocyte stress models. For further validation of *Coro2b* dependent cytoskeleton and focal adhesion related phenotypes, mouse embryonic fibroblast (MEF) from *Coro2b* knockout and wild type animals were isolated. Western blot analysis revealed CORO2B expression on protein level in MEFs and confirmed loss of CORO2B in respective knockout cells (supplemental Fig. 5a). Analysis for focal adhesion morphology showed enlarged mean focal adhesion size, but unaffected focal adhesion numbers per cell in respective *Coro2b* knockout MEFs (Fig. 6a–f). The specificity of this phenotype was confirmed by re-expression of CORO2B (Fig. 6c–e). In addition a modest increase in stress fiber density was observed in *KO* cells (supplemental Fig. 5). On a functional level, *Coro2b KO* results in a slightly impaired migratory behavior, but unaltered cellular spreading in MEFs (Fig. 6g–h and supplemental Fig. 5).

### CORO2B supports CFL1 recruitment towards focal adhesion sites and actin fibers

**Figure 7:**
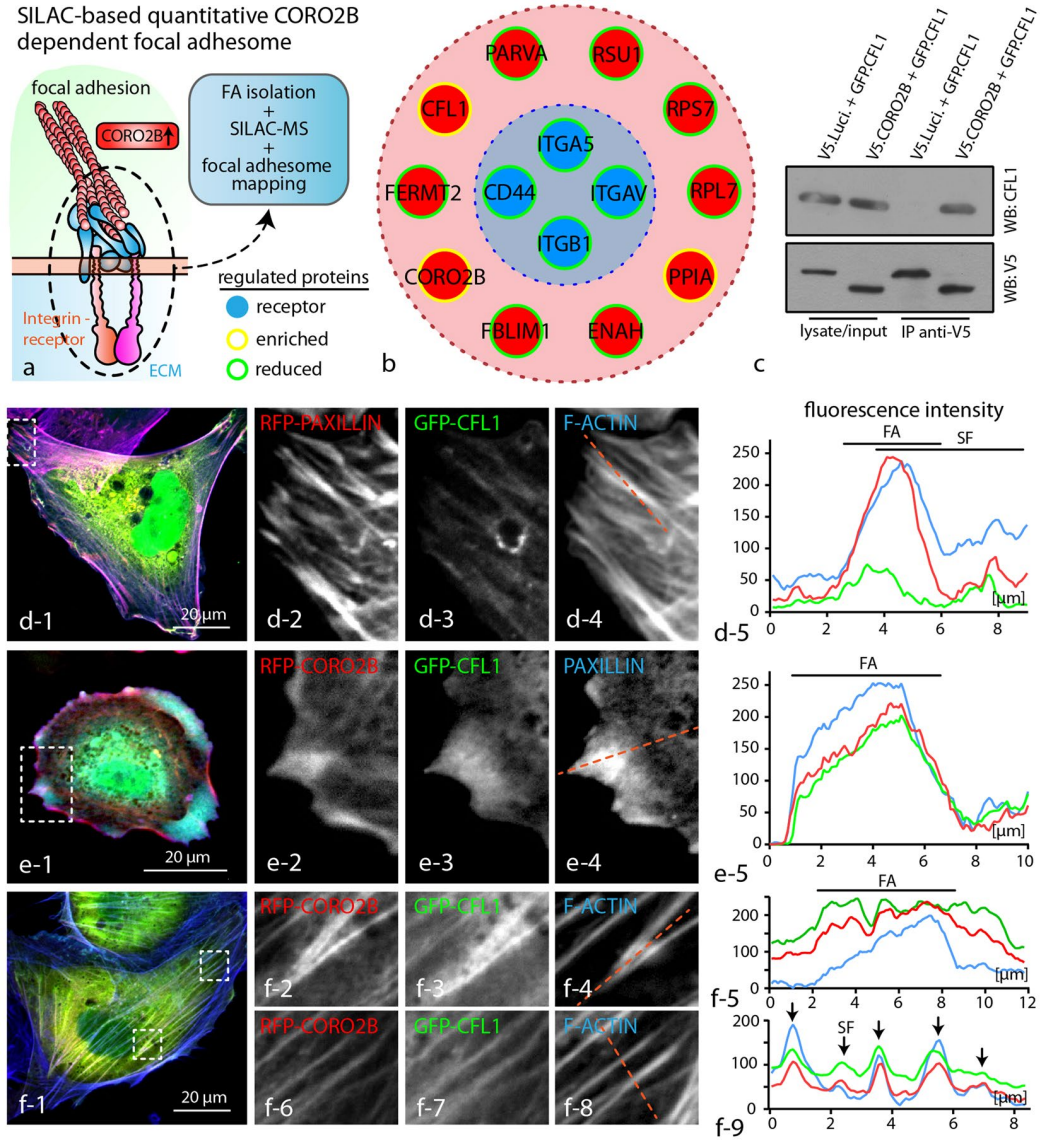
CORO2B increases CFL1 recruitment to focal adhesion sites and actin fibers. (**a**) Schematic illustrating the generation of a SILAC based quantitative CORO2B dependent focal adhesome by expression of CORO2B or GFP as control in human podocytes with subsequent focal adhesion isolation and mass spectrometry analysis. (See Methods section and Supplementary Dataset S1.) (**b**) Mapping of enriched and reduced proteins reveals impaired recruitment of the integrin receptors dimer ITGB1/ITGAV to CORO2B dependent focal adhesions. In contrast CORO2B and CFL1 recruitment to focal adhesion sites was increased. **(c)** CFL1 co-precipitates with CORO2B. V5-tagged CORO2B was immunoprecipitated by anti-V5 coated Sepharose beads. (**d–f**) Localization studies of fluorophore tagged CFL1 and CORO2B or PAXILLIN as control reveals CORO2B dependent recruitment of CFL1 to focal adhesions and fibrillary actin bundles. F-ACTIN was visualized by Phalloidin staining. Dotted lines indicate regions for line scan measurements of fluorescence intensities.

To uncover the mechanism of *Coro2b* dependent focal adhesion regulation we performed SILAC-based quantitative focal adhesion proteomics using CORO2B re-expressing human podocytes and control podocytes (Fig. 7a). Purification and enrichment of focal adhesions was basically performed by digestion of apical cell layers and subsequent isolation of ECM bound focal adhesions. SILAC-MS analysis of purified focal adhesions revealed an impaired recruitment of the integrin receptor dimers ITGB1/ITGAV as well as ITGA5 to focal adhesions sites (Fig. 7b and supplemental Fig. 6). Furthermore, also classical adaptor proteins such as PARVA, FERMT2 and FBLIM1 showed also decreased abundance levels (Fig. 7b). Surprisingly, the actin-severing factor CFL1 (together with CORO2B) was enriched in the focal adhesion fraction of *CORO2B* re-expressing podocytes (Fig. 7b). Expression of GFP tagged CFL1 confirmed weak basal CFL1 recruitment to focal adhesions and actin fibers in cultured human podocytes (supplemental Fig. 6a). To validate CORO2B dependent CFL1 recruitment to focal adhesions, co-expression experiments of fluorescence tagged CFL1 with either CORO2B or with PAXILLIN (as control) were performed. Line scans of focal adhesions and actin stress fibers confirmed indeed increased recruitment of CFL1 to these structures in the presence of CORO2B (Fig. 7d–f). These findings were furthermore supported by physical association of CFL1 to CORO2B as demonstrated by co-immunoprecipitation experiments (Fig. 7c). To examine alternative regulatory mechanisms for CFL1, immunoblotting and immunofluorescence staining for CFL1 and P-CFL1 (inactive form of CFL1) was performed using *Coro2b* knockout kidney sections and knockout cells. *Coro2b* knockout glomeruli and cells showed normal CFL1 expression and phosphorylation, indicating that CFL1 is not affected by other regulatory pathways in CORO2B deficient conditions (supplemental Fig. 6).

## Discussion

Precise regulation and modulation of the actin cytoskeleton/focal adhesion complex is indispensable for podocyte function and maintenance of the kidney filtration barrier^3^. Previous studies mainly focused on ubiquitously expressed key components of the focal adhesion and actin cytoskeleton, such as ITGB1, ILK, TALIN, MYO9, RHOA, RAC1 and CDC42^26–30^. Despite significant progress in our understanding of podocyte adhesion and cytoskeleton regulation, our knowledge regarding highly selectively expressed proteins is rather limited. In light of this, such a subset of specifically expressed proteins could be highly useful in the development of targeted therapeutic strategies or diagnostic biomarkers. Therefore, we re-analyzed available transcriptomic and proteomic datasets for podocyte specific expression of the coronin family of actin regulators^5,23^. This approach led to the identification of CORO2B as a novel, highly specifically expressed podocyte protein (Fig. 1).

Coronins represent a family of actin binding and regulating proteins defined by a unique type of WD40 domain^15,19^. Type 1 coronins were previously characterized as regulators for cell protrusion and leading-edge dynamics by modulating Arp2/3 mediated actin assembly and ADF/cofilin controlled actin severing/disassembly^17,19,20^. In 1999, CORO2B was first described and detected in neuronal cells, which was capable of directly binding F-actin and localizing to focal adhesions^21^. Aside from this very early report, CORO2B was completely neglected resulting in its unknown cellular function to date^14^.

Employing mRNA *in situ* hybridization, a LacZ reporter mouse model as well as immunofluorescence staining confirmed expression of *Coro2b* in the neuronal system and in the podocyte compartment, thereby validating our initial *omics* screen (Figs 1 and 2). *Coro2b* expression in podocytes was detectable from the capillary loop stage on (Fig. 2). At this developmental stage, endothelial as well as mesangial cells invaginate into the podocyte precursor compartment. This remarkable morphogenetic process initiates the formation of podocyte foot processes, which represent highly complex, mainly actin based cellular protrusions^31–34^. The importance of this phenotypic transition is also reflected by alterations in the podocyte transcription factor profile^32^. As the expression of CORO2B coincides with this essential maturational step, one could conclude that profound reorganization of the actin cytoskeleton and the focal adhesion interface is required to fully complete this morphogenetic transition. In line with this hypothesis, we detected CORO2B with a distinct localization pattern to filamentous actin and focal adhesions (Fig. 3).

Detailed analysis furthermore revealed the pronounced recruitment of CORO2B to the ventral actin cytoskeleton, representing a specialized subset of the general cytoskeleton. The term ventral actin cytoskeleton describes individual stress fibers with focal adhesions at both ends^24^. This specialized subset is involved in processes such as force sensing and generation – mechanisms potentially required in maintenance of podocyte foot processes and adhesion (supplemental Fig. 7). Given these functional analogies one could speculate that ventral stress fibers might resemble substructures of the complex cytoskeleton within podocyte foot processes. To finally test the *in vivo* role of CORO2B, a constitutive knockout mouse model was generated (Fig. 4). Surprisingly, no obvious general or glomerular phenotype was observed. Applying the Doxorubicin nephropathy model we tested for a potential role of *Coro2b* in podocyte stress conditions. Here, we observed that *Coro2b* knockout mice were partially protected and presented lower levels of proteinuria when compared to control wild type mice (Fig. 4j). Additional studies *in vitro* revealed that CORO2B initiates focal adhesion and ventral stress fiber disassembly in human podocytes (Fig. 5). Treatment with Doxorubicin drastically impaired dynamic spreading capacities of CORO2B expressing podocytes, correlating to our observations *in vivo* (Figs 4 and 5). Employing SILAC-based quantitative focal adhesion proteomics we aimed to identify the contribution of CORO2B to the composition of the focal adhesome. This approach helped us to uncover a potential explanatory mechanism, where CORO2B provokes recruitment of CFL1 to focal adhesions and actin stress fibers (Fig. 7).

CFL1 is extensively characterized in terms of its actin treadmilling by filament severing functions^35^. Type 1 Coronins titrate actin dynamics by regulating the Arp2/3 complex and CFL1 activity^19^. Remarkably, it was previously reported that the podocyte specific knockout of *Cfl1* leads to a disruption of the kidney filtration barrier in mice and even zebrafish^36,37^. In addition, CFL1 phosphorylation (indicating CFL1 inactivation) was observed in human glomerular disease samples and resembled cellular phenotypes of *Coro2b* knockout cell^37^. On the contrary, podocyte specific knockout for the GTPase CDC42 leads to severe proteinuria and at the same time respective podocytes exhibit a nearly complete loss of CFL1 phosphorylation (indicating CFL1 hyperactivation -^30^). Based on these observations and similar additional studies^35^, one could argue that CFL1 hypo- or hyper-activation might lead to podocyte dysfunction in a context dependent manner. The proposed mechanism for CFL1 recruitment by CORO2B towards focal adhesions does not interfere with upstream mechanisms for CFL1 activation or inactivation. In fact, actin de-polymerization factors like CFL1, CFL2 and ADF are highly redundant and tightly regulated by upstream signaling pathways^35^. Therefore, it is not surprising that *Coro2b* knockout mice do not exhibit an obvious *in vivo* phenotype as CFL1 activity might be tightly regulated. This hypothesis is supported by observations that re-expression of CORO2B as well as isolation of *Coro2b* knockout cells seems to overrule these compensatory mechanisms, leading to observable cellular phenotypes at least *in vitro* (Figs 5 and 6). A series of previous studies could establish the concept of balanced cytoskeletal dynamics as a prerequisite for podocyte function^30,38^. Especially, increased cytoskeletal dynamics were observed as a morphological and also causative correlate for podocyte disease. Interestingly, *Coro2b* knockout mice were partially protected towards Doxorubicin treatment, suggesting an attenuated increase of cytoskeletal dynamics in *Coro2b* knockout animals (Fig. 4). In line with this, we observed also increased dynamics and susceptibility to Doxorubicin treatment in CORO2B re-expressing podocytes (Fig. 5).

Given the highly specific expression of *Coro2b* in podocytes, the observation of a rather unaffected *in vivo* phenotype of knockout animals under physiological conditions raises the question for additional explanatory models: firstly, coronin proteins share structural homologies and might therefore compensate for a loss of CORO2B function. Remarkably, we did not detect any overt alterations in terms of abundance or composition in proteomics studies on either wild type or knockout cells (supplemental Fig. 8 and dataset S2). This finding does not completely exclude compensatory events, but suggests that signaling or localization might be more relevant in this context (as also shown for CFL1). Secondly, we also cannot exclude a background dependent penetrance of the *Coro2b* knockout phenotype. Previous studies revealed for a series of prominent genes context dependent penetrance and expressivity, or even no observable phenotypes in human loss of function mutations as well as knockouts in animal models^39–42^. Incomplete penetrance and expressivity was also observed for monogenetic causes of familiarly nephrotic syndromes^43–45^. Therefore, future studies might reveal context dependent factors influencing *Coro2b* function in glomerular podocytes.

**Figure 8:**
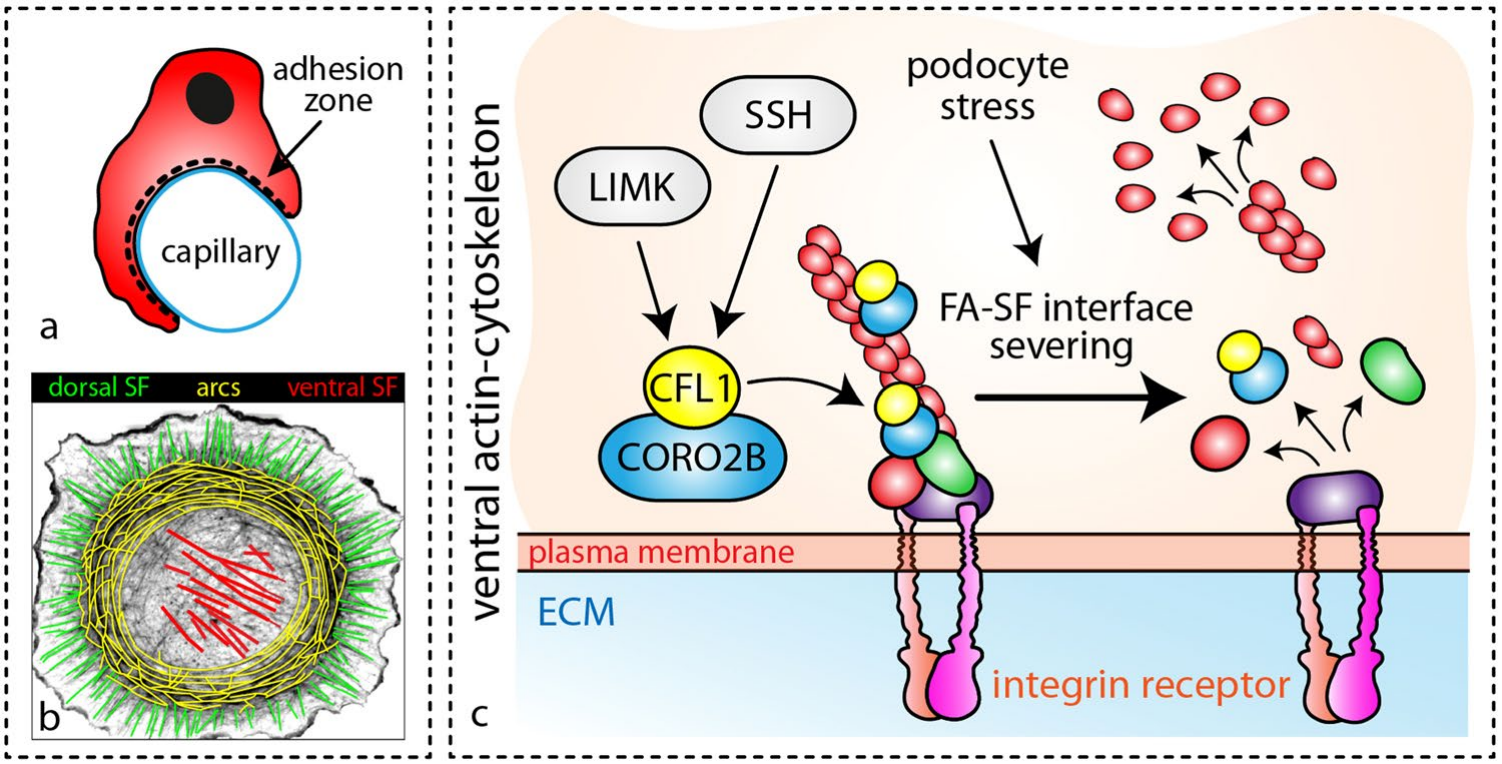
CORO2B is a localization module for CFL1 to the ventral actin cytoskeleton and a prerequisite for CFL1 mediated actin severing.

In summary, we provide on the basis of the identification of CORO2B as a novel podocyte specific cytoskeleton protein a new concept, describing a cell-inherent mode of focal adhesion/filamentous actin interface regulation by CORO2B dependent recruitment of CFL1 (Fig. 8).

## Methods

### Animals

Mice carrying *Coro2b* targeted alleles were generated by injection of KOMP-generated ES cells (EPD0392_7_C01, cells were purchased from KOMP, UC Davis, USA) into C57Bl6 blastocysts. F1 progeny and subsequent generations were genotyped by PCR standard conditions to detect different allele configurations using following primers: *mCoro2b* wildtype allele: (fp) 5′-GGT TCC TGG AAT CTG ACT CAG GGC TTC-3′ and (rp) 3′-CAG GGA CAG GGA CTA GAG GGA CA-5′; for *mCoro2b* knockout allele: (fp) 5′-TGA AAC CCT GAG TTC AGT CCC C-3′ and (rp) 3′-GCC TGA ATA TTC TAG TCC CGA CAC-5′; and for *mCoro2b-lacZ* allele: (fp) 5′-GGG ATC TCA TGC TGG AGT TCT TCG-3′ and (rp) 3′-GCC TGA ATA TTC TAG TCC CGA CAC-5′. Respective offspring was backcrossed for 6 generations on a C57BL6 (SV129/C57BL6-mixed) genetic background. In respective stress experiments, mice at an age of 6–8 weeks were challenged using Doxorubicin according to standard protocols^46^. Reporter strains for isolation of primary podocytes was established using Nphs2Cre* Gt(ROSA)26Sor ^*tm4(ACTB-tdTomato,-EGFP)Luo/J*^ mice (purchased from JAX mice). All animal experiments were performed in accordance to the German law for the welfare of animals, the NIH Guide for the care and use of laboratory animals and were approved by the Regierungspräsidium Freiburg, Germany. Housing and breading of the animals was performed in a specific pathogen free facility according to standard procedures. They were kept at 12 hour day/night cycle and had free access to water and chow.

### *In situ* hybridization

All primers used in this study for generation of *in situ* probes are collectively described in supplementary dataset S3. P1 mouse kidneys served as source for whole mRNA extracts and this were used as trampled for RT-PCR and cloning of fragments of the coding sequence and 3′-non coding region. To generate sense and antisense digoxigenin-labeled probes (digoxigenin RNA labeling mix; Roche Applied Science, Mannheim, Germany), PCR products were cloned into pBluescript II KS (-), linearized and transcription was done by use of T3 and T7 RNA polymerases (Promega, Mannheim, Germany). Kidneys at p1, E16.5 and embryos at E14.5 were fixed overnight at 4 °C in 4% paraformaldehyde, paraffin embedded and sectioned to 8 μm slides. Slides were then treated with proteinase K, re-fixed with 4% paraformaldehyde and were acetylated by using acetic anhydride (0,25% acetic anhydride in 0,1 M triethanolamine (T-1377; Sigma, Schnelldorf, Germany). Hybridization was done at 68 °C in hybridization buffer (50% formamide, 5 × SSC, yeast RNA (50 g/ml), 1% SDS, heparin (50 g/ml), 0,1% probe). Stringency washes were performed with wash I (50% formamide, 5 × SSC (pH4,5), 1%SDS) and wash II (50% formamide, 2 × SSC). For mRNA labeling, slides were incubated with alkaline phosphatase-conjugated anti-digoxigenin antibody 1:3000 at 4 °C overnight followed by BM purple staining (Roche Applied Science, Mannheim, Germany). For digital imaging acquisition an Axioplan2 microscope (Zeiss, Oberkochen, Germany) was used.

### Immunogold TEM procedures

For transmission electron microscopy kidney samples were fixed using 4% PFA in PB by perfusion via A. renales using small silicone catheters. After perfusion fixation the kidneys were removed and sliced into 50 micrometer thin vibratome sections. Immunogold pre-embedding labeling was performed as described earlier^47^. Briefly anti-CORO2B antibody was incubated overnight at 4 °C, sections were washed in PB and incubated overnight in secondary antibody at 4 °C (1.4 nm nanogold, Nanoprobes Inc., NY, USA). Gold labeling was enhanced using HQsilver kit (Nanoprobes Inc., NY, USA). Finally sections were embedded in Durcupan resin (Sigma-Aldrich, Germany) and ultrathin (40 nm) sections were cut using a Leica UC6 ultracut. Sections were imaged using a Philips CM 100 TEM.

### Expression of *Coro2b* constructs in immortalized human podocytes

Transfection of immortalized human podocyte cells (kindly provided by M. Saleem, University of Bristol, UK) was performed using Amaxa nucleofector technology (Lonza, Basel, Switzerland) according to manufacturer´s instructions. Constructs for mouse Coro2b and GFP-Cofilin1 were purchased (OriGene Technologies, Rockville, USA). The immortalized cell line represents the most used cell line for glomerular research^48^. All experiments were carried out in accordance with guidelines of the University Medical Center Freiburg, and were approved by the Regierungspräsidium Freiburg, Germany (this applies also to all other cell culture/*in vitro* studies within this project). N or C-terminal tagging of constructs was archived by cloning of Coro2b into V5, GFP or mCherry containing Vectors. Constructs for mCherry-Paxillin was kindly provided by C. Waterman, (National Heart, Lung, and Blood Institute, Bethesda, USA).

### Isolation of mouse embryonic fibroblasts

Isolation of mouse embryonic fibroblast (MEF) cells was performed according to standard procedures as described previously^49^. Briefly, embryos at E9.5–10.5 were freshly harvested and maternal tissues, the head and all innards were removed. The remaining tissue was dissected with a razor blade and digested by adding of trypsin for 30 minutes at 37 °C. MEF culturing medium (DMEM, 10% FBS, Penicillin/Streptomycin) was added and the tissue was further dissolved by pipetting 20 times. Then the suspension was seeded into cell culture flask, MEFs were expended for 3 days and experiments were performed between P3-P5.

### Antibodies

All antibodies used in this study are collectively described in Supplementary Dataset S3.

### Immunoprecipitation

Co-immunoprecipitation was performed as described previously^5^. Briefly, HEK 293 T cells were transfected with 4 µg DNA of the indicated constructs and cells were incubated for 24 h. The PEI (Polyethylenimin) method was used for transiently transfection of HEK cells (HEK293T cells were purchased from the ATCC cell repository, USA). Cells were lysed in 1% Triton X-100 lysis buffer (1% Triton X-100, 20 ml Tris-HCL, 50 mM NaCL, 50 mM NaF, 15 mM Na_4_P_2_O_7_, 1 mM EDTA, pH 7.4) for 30 min at 4 °C. Cell lysates were centrifuged (15,000 × *g*; 15 min; 4 °C) and the supernatant was incubated with 1 µg mouse anti-V5-tag antibody (MCA1360, Serotec) for 12 h at 4 °C. Thereafter cell lysates were incubated with 20 μl of protein G-Sepharose beads for 1 h at 4 °C. Sepharose beads were washed 5 times with lysis buffer to remove unbound proteins. Finally beat-bound proteins were resolved in *Laemmli* sample buffer (95 °C, 5 min) and analyzed by standard SDS-polyacrylamide gel electrophoresis based western blotting technique.

### Histology

Dissection and fixation of mouse kidneys was described previously^34^. In brief, kidneys from mice younger than p7 were dissected under a binocular light microscope and the kidney capsule was removed. For fixation, kidneys were incubated in 4% PFA in PBS at 4 °C for 12 h. Kidneys from mice older than p7 were fixated by perfusions per kidney of 4–5 ml 4% PFA in PBS via the A. renales. Then, capsules were removed and kidneys were immersion fixated in 4% PFA at 4 °C for 12 h. Dehydration and embedding in paraffin was performed using an automated system (Histokinette, Leica, Germany). Paraffin embedded kidneys were cut in 8 μm sections using a Leica microtome (Leica, Germany) and subsequent Hematoxylin-Eosin and Periodic-acid-Schiff staining procedures were performed by the Department of Pathology, University Hospital of Freiburg. For image acquisition was an Axioplan 2 microscope (Zeiss, Germany) used. Human kidney samples from unaffected areas of tumor nephrectomies were used and use of this samples was approved by the Scientific-Ethical Committee of the University Medical Center of Freiburg.

### Immunofluorescence and LacZ staining of kidney sections

Immunofluorescence studies were performed as previously described^50^. In brief, snap frozen tissue samples of mice kidneys were cut in 4 µm cryosections using a cryotome (Leica, Wetzlar, Germany). Cryosections were subsequently fixated using 4% PFA in PBS for 3 minutes at room temperature. Samples were blocked with 5% BSA (Sigma, Schnelldorf, Germany) diluted in PBS and incubated with primary antibodies, each step for 1 hour at room temperature. Sections were 3 times washed with PBS and fluorophore-conjugated secondary antibodies (Invitrogen, Karlsruhe, Germany) were applied for 45 minutes. Slides were extensively washed with PBS and mounted using Prolong Gold Antifade (Invitrogen, Darmstadt, Germany). Image acquisition was done using a Zeiss Axioscope 40FL microscope equipped with an Axiocam MRc5 digital video camera and conventional HBO lamp (Carl Zeiss, Oberkochen, Germany). Use of human kidney biopsy material was approved by the Scientific-Ethical Committee of the University Medical Center of Freiburg. Kidney samples were from unaffected areas of tumor nephrectomies. Staining procedure for the LacZ reporter was performed according to standard procedures. In brief, kidneys of *Coro2b* heterozygous mice expressing the β*-*Galactosidase reporter molecule downstream of the *Coro2b* promoter were snap frozen and cut in 10 µm cryosections using a cryotome. Subsequently, kidney sections were fixed in 0.2% glutaraldehyde (0.1 M sodium phosphate buffer, 5 mM EGTA, 2 mM MgCl2, pH 7.3). β*-*Galactosidase staining with X-gal staining solution (1 mg/mL X-gal (Sigma), 5 mM potassium ferrocyanide (Sigma), 5 mM potassium ferricyanide (Sigma) was performed over night at 37 °C. Sections were extensively washed in wash buffer (0.1 M Sodium phosphate buffer, pH 7.3, 2 mM MgCl2, and 0.01% sodium deoxycholate) followed by post*-*fixation and mounting. For image acquisition an Axioplan 2 microscope (Zeiss, Germany) was used.

### Measurement of urinary albumin and creatinine

Measurement of urinary albumin and urinary creatinine was performed using a mouse specific fluorescent based kit for albumin (Progene, Germany) and using an enzymatic kit for creatinine (Creatinine PAP LT-SYS, Labor&Technik, Eberhard Lehmann GmbH, Germany). Albumin and creatinine in was measured in spot urine samples from wild type and knockout mice and levels of proteinuria were expressed as albumin to creatinine ratio.

### Isolation of primary podocytes

Isolation of podocytes was performed as previously described^34^. Kidneys were cut in small pieces and mixed into digestion buffer (1 mg/ml Collagenase, 1 mg/ml Proteinase, 50 U/ml DNase in 1xHBSS, 37 °C). The solution was incubated at 37 °C for 7 min and further dissected by pipetting. After incubation the solution was rubbed through a 100 µm cell strainer using a stamp of a 5 ml syringe and the strainer was carefully washed using ice cold 1xHBSS. The flow-through was now filtered through a 70 µm cell strainer and washed again with 1xHBSS. Glomeruli were now collected from the flow-through by filtering through a 70 µm cell strainer and were washed from this cell strainer using 1xHBSS. The now obtained glomeruli were centrifuged at 4 °C, 2000 g for 10 min and the pellet was dissolved in primary podocyte medium (RPMI medium supplemented with 10% FBS, Penicillin/Streptomycin, ITS). Finally the glomeruli were seeded into Collagen IV coated cell culture flask and cultured at 37 °C and 5% CO_2._ Glomerular cells were grown out for 7 days and FACS sorted to separate the GFP labeled podocyte fraction from the non-podocyte fraction.

### Immunofluorescence on cultured cells

Cells were cultured on Collagen IV coated glass coverslips for 24 h before staining. Cells were fixated using 4% PFA in PBS for 10 minutes at room temperature. PFA was quenched by application of 50 mM NH_4_CL, followed by permeabilization with 0.1% Triton-X-100 in PBS for 3 minutes and blocking in 5% BSA (Sigma, Schnelldorf, Germany) diluted in PBS for 1 h. After clocking cells were incubated with primary antibodies for 1 hour at room temperature. Coverslips were 3 times washed with PBS and fluorophore-conjugated secondary antibodies (Invitrogen, Karlsruhe, Germany) were applied for 45 minutes. The staining procedure was followed by mounting in Prolong Gold antifade (Invitrogen, Schnelldorf).

### Single cell migration, cellular spreading and live cell imaging

Measurement of single cell migration was performed on ibidi μ-treat dishes (ibidi, Martinsried, Germany) using a Nikon Biosstation IM device (Nikon, Düsseldorf, Germany). Analysis of single cell migration was done by using the ManualTracking and ChemoTaxis plugin for FIJI NIH ImageJ 1.46.

For cell spreading assays cells were seeded on Collagen IV (50 µg/ml, Sigma, Schnelldorf, Germany) pre-coated cover-slips for indicated time points. For experiments assaying Doxorubicin (pharmacy of University Hospital Freiburg) or protramine sulfate ((Sigma, Schnelldorf, Germany)) treatment, cells were pre-treated with 1 µg/ml Doxorubicin for 48 hours or 300 µg/ml protamine sulfate for 10 minutes. Cells on cover-slips were fixed in 4% PFA in PBS and cells were stained for F-ACTIN (Phalloidin) or PAXILLIN. Image acquisition was done using a Zeiss Axioscope 40FL microscope with a 20x objective. Images were analyzed using FIJI NIH ImageJ 1.46.

Live cell imaging was performed after transfection of the indicated constructs and seeding on ibidi μ-treat dishes using a Zeiss Cell Observer TIRF microscope, equipped with a Alpha-Plan-Apochromat 100x objective.

### Evaluation of focal adhesion, stress fiber morphology and fluorescence intensity

Measurement of focal adhesion size and distribution was basically performed as described previously^51^. In brief, cells were seeded on Collagen IV (50 µg/ml, Sigma, Schnelldorf, Germany) pre-coated cover-slips and were stained for the focal adhesion component PAXILLIN and for F-ACTIN by use of Phalloidin as described above. Image acquisition was done using a Zeiss Axioscope 40FL microscope with a 63x objective and using standardized exposure times. Quantification of focal adhesion characteristics was performed with a custom written macro embedded in FIJI NIH ImageJ 1.46. Evaluation of F-Actin was also performed by using FIJI NIH ImageJ 1.46. Phenotyping of cells was done by individual assessment of cells for 3 criteria (a: intact central focal adhesions (FA) and actin cytoskeleton (AC) b: disassembling/reduced central FA and AC; c: collapsing/loosed FA and AC). For quantification of CORO2B re-expressing podocytes, GFP-CORO2B expressing cells were identified and selected for strong GFP signal intensity. Image acquisition was done using a Zeiss Axioscope 40FL microscope with a 63x objective and using standardized exposure times. For analysis of fluorescence intensity, cells were cultured and stained with the indicated antibodies as described above. Representative line scans were performed using the plot profile tool FIJI NIH ImageJ 1.46.

### Focal adhesion complex isolation

The SILAC labeling of human immortalizes podocytes for quantitative MS analysis was previously described^5,52^. Isolation of focal adhesion complexes was performed as previously described^5^. In brief, CORO2B-GFP or GFP expressing human immortalized podocytes were seeded to a 15 cm cell culture dish and focal adhesion proteins were crosslinked by application of DSP (3,3′-Dithiobis(sulfosuccinimidylpropionate); 0.5 mM; Sigma-Aldrich) and DPPB (1,4-Bis[3-(2-pyridyldithio)propionamido]butane; 0.05 mM; Sigma-Aldrich) for 10 minutes. Podocytes were washed with PBS and cross-linkers were quenched using 1 M Tris-HCl (pH 8, 10 min). Podocyte cell bodies were removed via application of hydrodynamic force to the culture dish using a waterpik (2 × 10 s; PBS). Culture dish/ECM bound focal adhesion complexes remained and were solubilized by scraping in 100 μl scraping buffer (125 mM Tris-HCl (pH 6.8), 1% (w/v) SDS, 15% (v/v) β-mercaptoethanol). Samples were denatured at 70 °C for 10 min by adding DDT and Western blotting or MS analysis was performed subsequently. MS analysis of isolated focal adhesion complexes was performed at the proteomics core facility of the University of Freiburg. Only proteins detected in both SILAC-MS replicates were used for further analysis. This candidate list was subsequently filtered for proteins annotated to the GO-Term „focal adhesion [GO:0005925]“ and ranked according to CORO2B dependent enrichment. Proteins were considered as enriched/upregulated with a log2 ratio CORO2B/control >0.4 or as downregulated/reduced with a log2 ratio <−0.4. (See Supplementary Dataset S1.)

### Proteome profiling of MEF cells

Isolation and culture of MEF cells was performed as described above. Five splitting cycles after isolation, MEF cells were used for whole cell proteomics profiling. For quantitative proteomic analyses, sample preparation and mass spectrometry analysis (Q-Exactive plus system, Thermo Scientific, Bremen, Germany) were performed as reported previously^53,54^. LC-MS/MS data analysis was performed as reported before^54^. The UniProt database was used for Gene Ontology (GO) term annotation^55^. In addition, the consensus integrin adhesome was used to filter for core focal adhesome components^56^. (See also supplementary dataset S2.)

### Statistics and reproducibility

If not stated otherwise, data are expressed as mean ± SEM. Paired Student´s t-test or ONE-WAY Anova (multiple comparison test - Tukey) were used based on data distribution. Statistical significance was defined as *p < 0.05, **p < 0.01, ***p < 0.001 and ****p < 0.0001, n.s. - not significant. Number of independent experiments and total amount of analyzed cells are stated in the figures.

## Electronic supplementary material

Supplementary Figures

Dataset 1

Dataset 2

Dataset 3
