# Supplementary material for: The WD40-domain containing protein CORO2B is specifically enriched in glomerular podocytes and regulates the ventral actin cytoskeleton
Source: Sci Rep. 2017 Nov 21;7:15910. doi: 10.1038/s41598-017-15844-1 (PMC5698439; doi:10.1038/s41598-017-15844-1)
Supplement: Supplementary file 1 — Supplementary Figures [file 41598_2017_15844_MOESM1_ESM.pdf]

The WD40-domain containing protein CORO2B is specifically enriched in glomerular podocytes and regulates the ventral actin cytoskeleton

Rogg M[1], Yasuda-Yamahara M[1,2], Abed A[1], Dinse P[1], Helmstädter M[1], Conzelmann AC[1], Frimmel J[1], Sellung D[1], Biniossek ML[3], Kretz O[1,4,5], Grahammer F[1,5], Schilling O[3,6,7], Huber TB[1,5,6,#] and Schell C[1,8,9]

[1] Department of Medicine IV, Medical Center – University of Freiburg, Faculty of Medicine, University of Freiburg, Germany

[2] Department of Medicine, Shiga University of Medical Science, Otsu, Shiga, Japan

[3] Institute of Molecular Medicine and Cell Research, University of Freiburg, Freiburg, Germany

[4] Institute of Anatomy and Cell Biology, Dept. for Neuroanatomy, Medical Faculty, Albert-Ludwigs-University Freiburg, Freiburg, Germany

[5] III. Department of Medicine, University Medical Center Hamburg-Eppendorf, Hamburg, Germany

[6] BIOSS Center for Biological Signalling Studies and Center for Systems Biology (ZBSA), Albert-Ludwigs-University, Freiburg, Germany

[7] German Cancer Consortium (DKTK) and German Cancer Research Center (DKFZ), Heidelberg, Germany

[8] Institute for Surgical Pathology, University Medical Center Freiburg, Germany

[9] Berta-Ottenstein Programme, Faculty of Medicine, University of Freiburg

# To whom correspondence should be addressed:

Tobias B. Huber
III. Department of Medicine
University Medical Center Hamburg-Eppendorf
Martinistr. 52, D-20246 Hamburg, Germany
t.huber@uke.de

Supplementary Figures

Figure S1

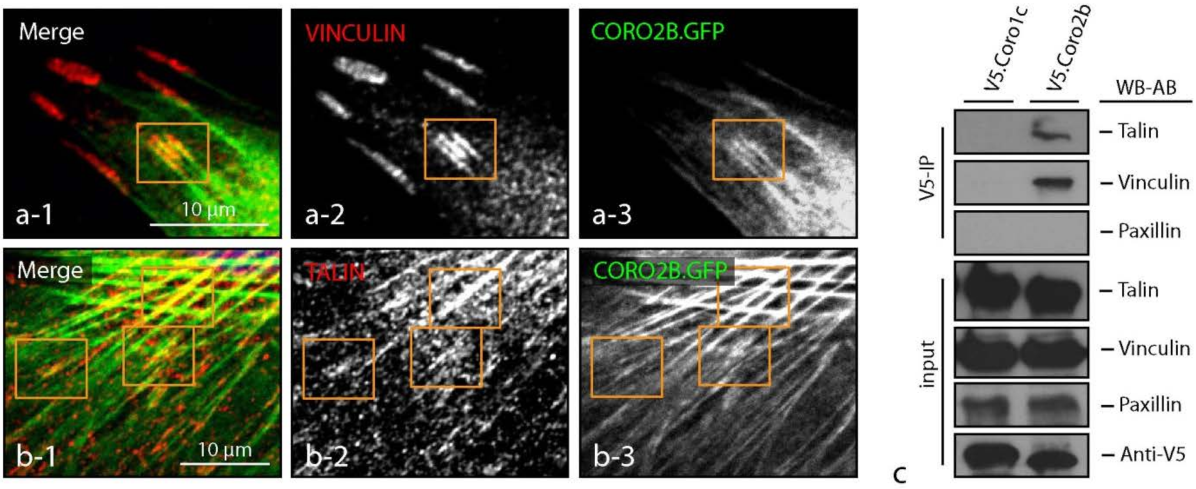

Figure S1. CORO2B associates with the focal adhesion component VINCULIN and TALIN.

(**a-b)** Immunostaining for VINCULIN and TALIN in CORO2B-GFP expressing human podocytes shows overlapping localization at focal adhesions. **(c)** Immunoprecipitation of V5-tagged CORO2B co- precipitates the focal adhesion components VINCULIN and TALIN but not PAXILLIN.

Figure S2

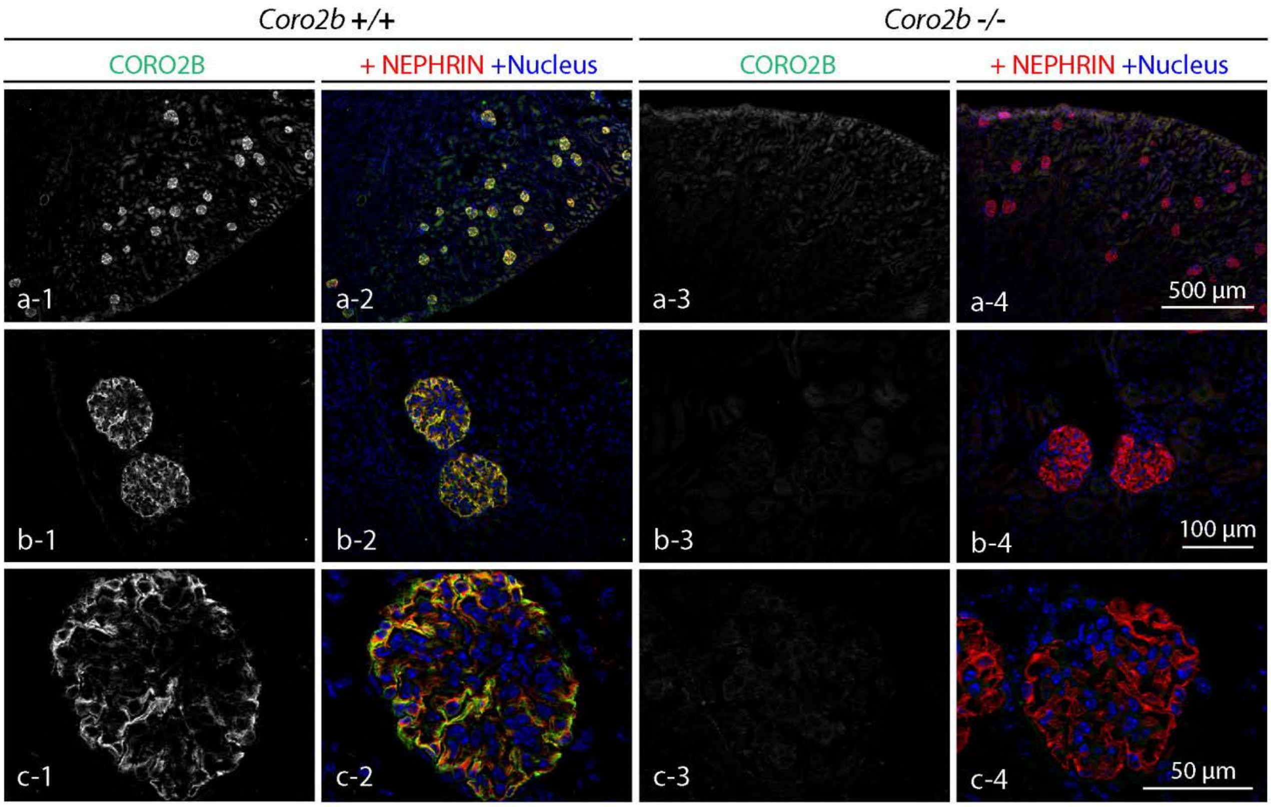

Figure S2. CORO2B immunofluorescence staining confirms Coro2b knockout.

(a-c) Cryo-slides of *Coro2b-KO* and *WT* kidneys showing specific expression of CORO2B in podocytes of *WT* animals and confirms loss of CORO2B expression in *KO* animals. The podocyte compartment was visualized by co-staining for the slid diaphragm component NEPHRIN.

Figure S3

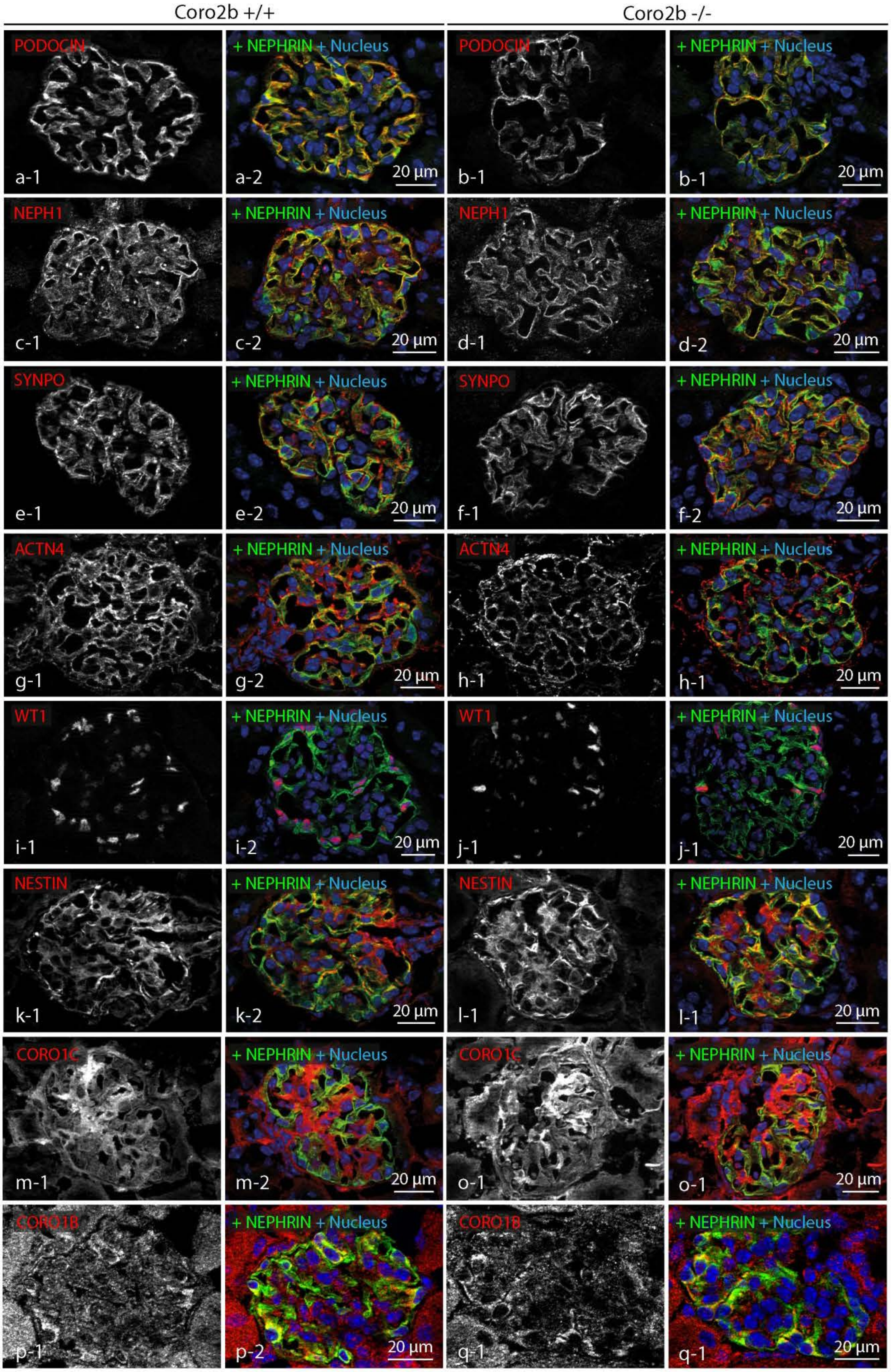
Coro2b +/+
Coro2b -/-
PODOCIN
+ NEPHRIN + Nucleus
a-1
a-2
b-1
b-1
NEPH1
c-1
c-2
d-1
d-2
SYNPO
e-1
e-2
f-1
f-2
ACTN4
g-1
g-2
h-1
h-1
WT1
i-1
i-2
j-1
j-1
NESTIN
k-1
k-2
l-1
l-1
CORO1C
m-1
m-2
o-1
o-1
CORO1B
p-1
p-2
q-1
q-1
20 μm

Figure S3. *Coro2b knockout* does not affect expression and distribution of podocyte specific proteins.

(a-o) Analysis of NEPHRIN, PODOCIN, NEPH1, SYNPO, ACTN4, WT1, NESTIN, CORO1C and CORO1B expression in *wild type* and *Coro2b knockout* podocyte reveals no difference with regard to expression pattern and distribution of slit-diaphragm as well as cytoskeleton proteins. Immunofluorescence staining was performed on cryosections of KO or WT animals by use of the indicated antibodies. Nuclei were counterstained by DAPI blue.

Figure S4

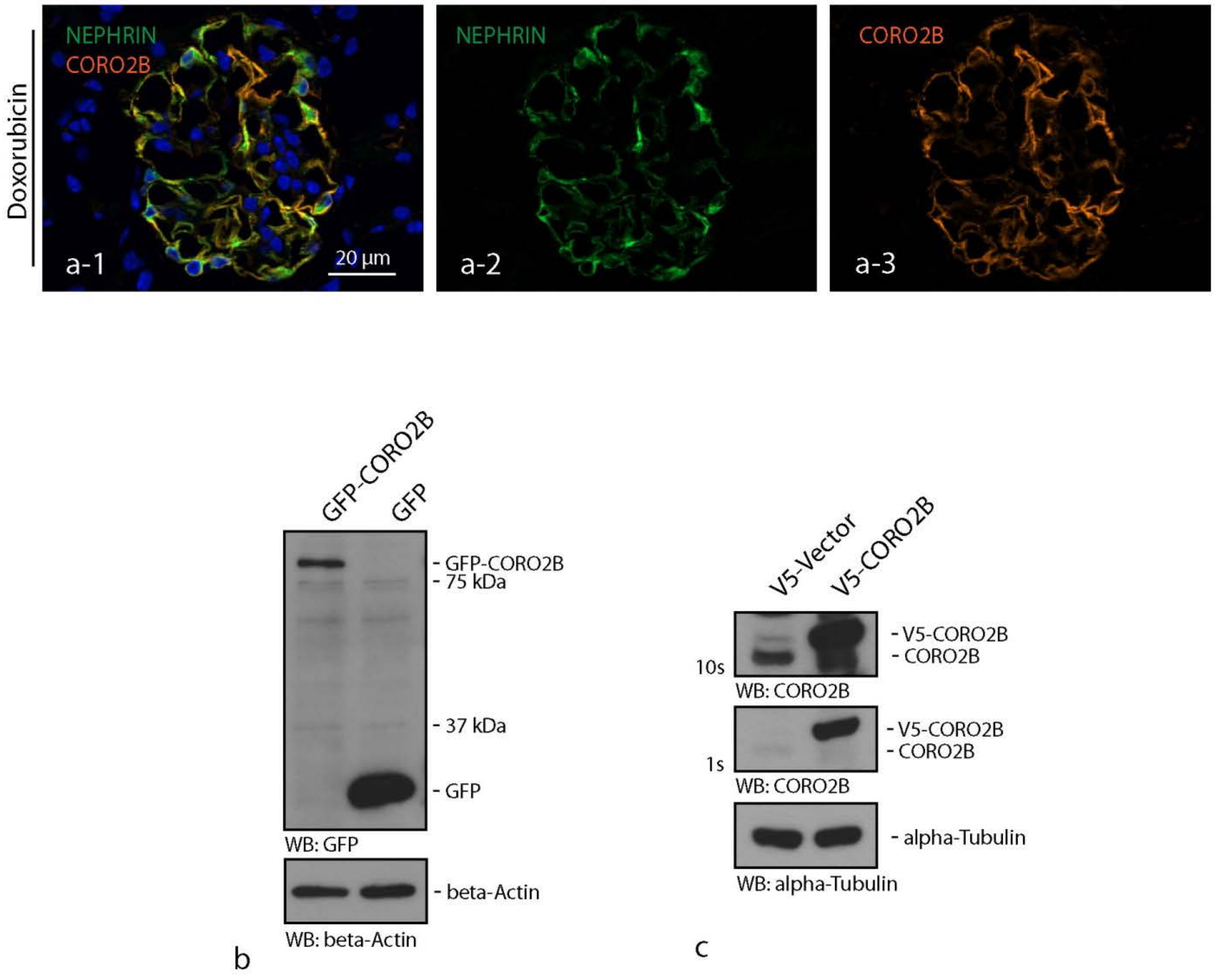

Figure S4. Confirmation of CORO2B expression.

(a) CORO2B expression is stable in Adriamycin treated mice. **(b-c)** Western blot for GFP and CORO2B confirmed expression of CORO2B in transfected human podocytes.

Figure S5

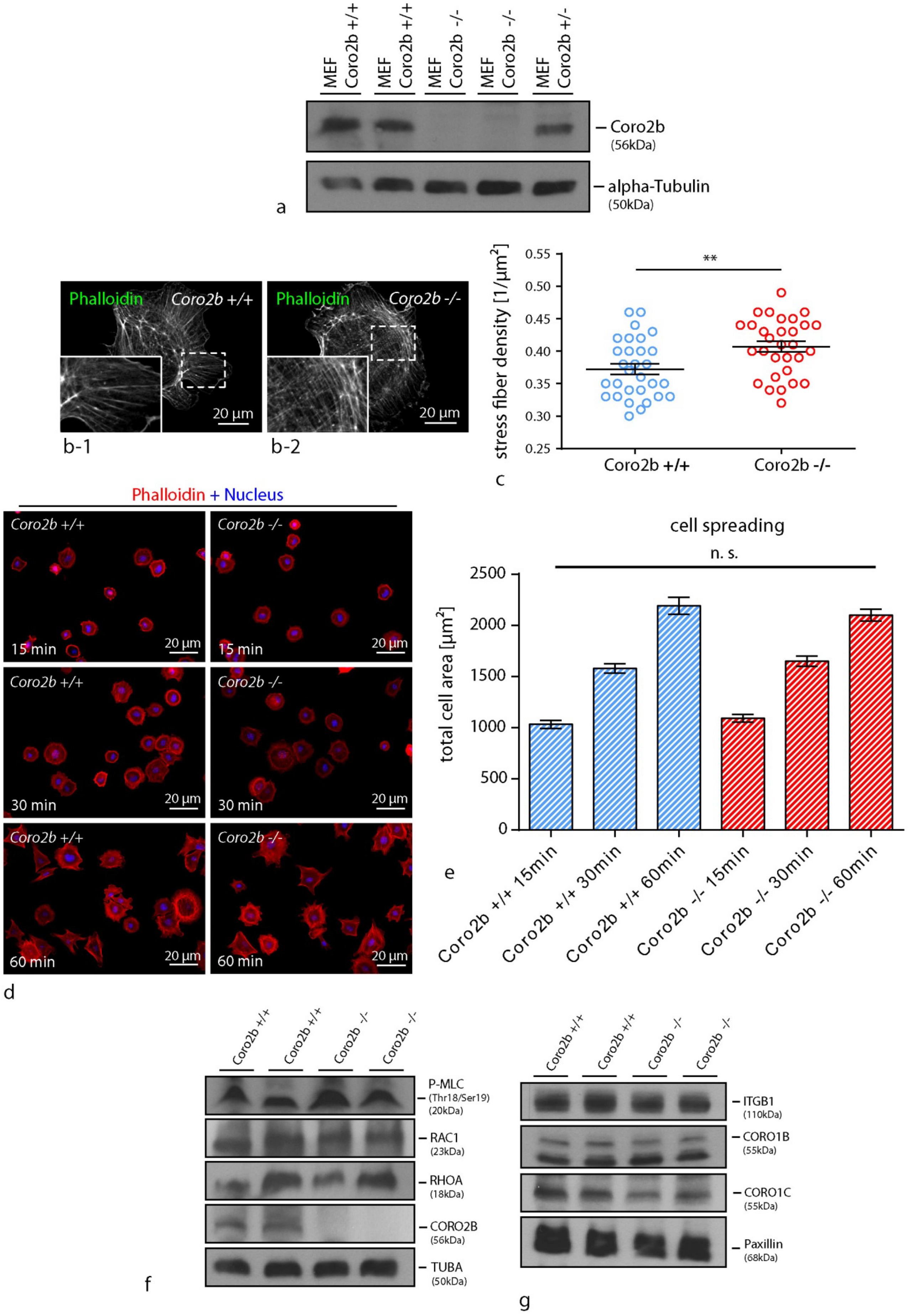
MEF Coro2b +/+
MEF Coro2b +/+
MEF Coro2b -/-
MEF Coro2b -/-
MEF Coro2b +/-
Coro2b (56kDa)
alpha-Tubulin (50kDa)
a
Phalloidin
Coro2b +/+
Coro2b -/-
20 µm
b-1
b-2
stress fiber density [1/µm^2]
0.55
0.50
0.45
0.40
0.35
0.30
0.25
**
Coro2b +/+
Coro2b -/-
c
Phalloidin + Nucleus
Coro2b +/+
Coro2b -/-
15 min
30 min
60 min
20 µm
d
cell spreading
n. s.
total cell area [µm^2]
2500
2000
1500
1000
500
0
Coro2b +/+ 15min
Coro2b +/+ 30min
Coro2b +/+ 60min
Coro2b -/- 15min
Coro2b -/- 30min
Coro2b -/- 60min
e
Coro2b +/+
Coro2b +/+
Coro2b -/-
Coro2b -/-
P-MLC (Thr18/Ser19) (20kDa)
RAC1 (23kDa)
RHOA (18kDa)
CORO2B (56kDa)
TUBA (50kDa)
f
ITGB1 (110kDa)
CORO1B (55kDa)
CORO1C (55kDa)
Paxillin (68kDa)
g

Figure S5. Cellular spreading capability of *Coro2b knockout* MEFs is not impaired.

(a) Western blot for CORO2B reveals expression of CORO2B in MEF cells and confirms loss of CORO2B expression in MEFs from respective *Coro2b knockout* animals. **(b-c)** Stress fiber density was increased in *Coro2b knockout* MEFs (p=30 WT and 30 KO cells out of 3 independent experiments were analyzed: ** p<0.01). **(d-e)** Cell spreading of *Coro2b knockout* MEFs on collagen IV coated glass cover slips was not impaired (n=254 to 302 cells out of 3 independent experiments were analyzed per condition; n.s – non significant). **(f-g)** Western blot experiments in MEFs (wild type vs. *Coro2b* knockout) could not detect differences in expression levels of assessed cytoskeleton regulators or focal adhesion components.

Figure S6

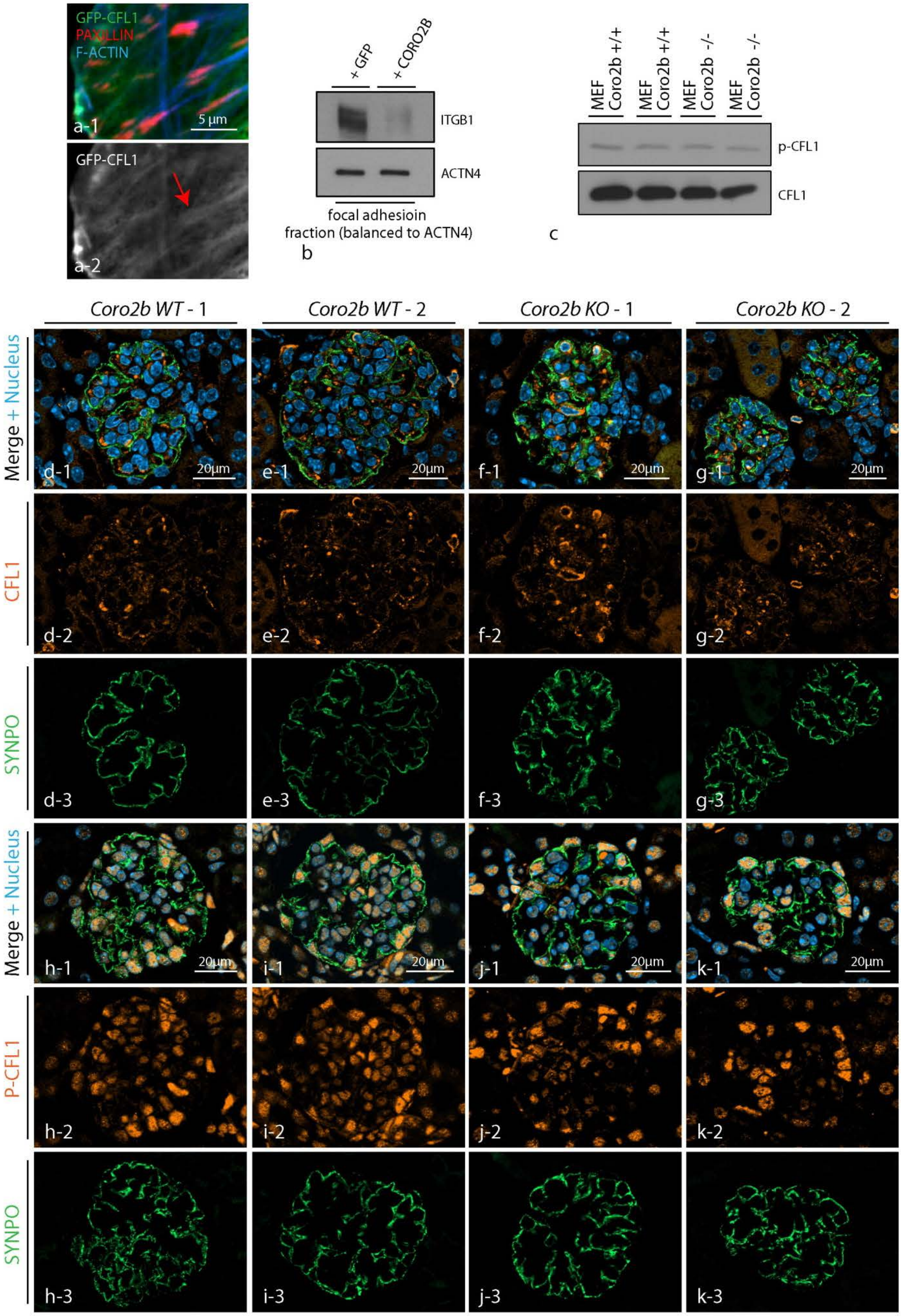
GFP-CFL1
PAXILLIN
F-ACTIN
5 µm
a-1
GFP-CFL1
a-2
+ GFP
+ CORO2B
ITGB1
ACTN4
focal adhesioin
fraction (balanced to ACTN4)
b
MEF Coro2b +/+
MEF Coro2b +/+
MEF Coro2b -/-
MEF Coro2b -/-
p-CFL1
CFL1
c
Coro2b WT - 1
Coro2b WT - 2
Coro2b KO - 1
Coro2b KO - 2
Merge + Nucleus
CFL1
SYNPO
P-CFL1
20µm
d-1
e-1
f-1
g-1
d-2
e-2
f-2
g-2
d-3
e-3
f-3
g-3
h-1
i-1
j-1
k-1
h-2
i-2
j-2
k-2
h-3
i-3
j-3
k-3

Figure S6. CFL1 expression and phosphorylation levels are not regulated in *CORO2B knockout* animals and cells.

(a) Expression of GFP tagged CFL1 in human podocytes reveals weak localization to focal adhesions and actin fibers. F-ACTIN was visualized by Phalloidin and focal adhesions by PAXILLIN staining. **(b)** Western blot of isolated focal adhesions confirm reduced recruitment of ITGB1 to CORO2B dependent focal adhesions. **(c)** Western blot analysis of isolated *Coro2b-KO* and *WT* MEFs reveals normal expression and phosphorylation of CFL1 in *Coro2b knockout* MEFs. **(d-k)** Analysis of CFL1 expression and phosphorylation in *wild type* and *Coro2b knockout* glomeruli shows no difference with regard to expression abundance and distribution of CFL1 or p-CFL1. Paraffin embedded kidney sections were stained for CFL1 or p-CFL1, and SYNPO to label the podocyte compartment. Nuclei were stained by DAPI blue.

Figure S7

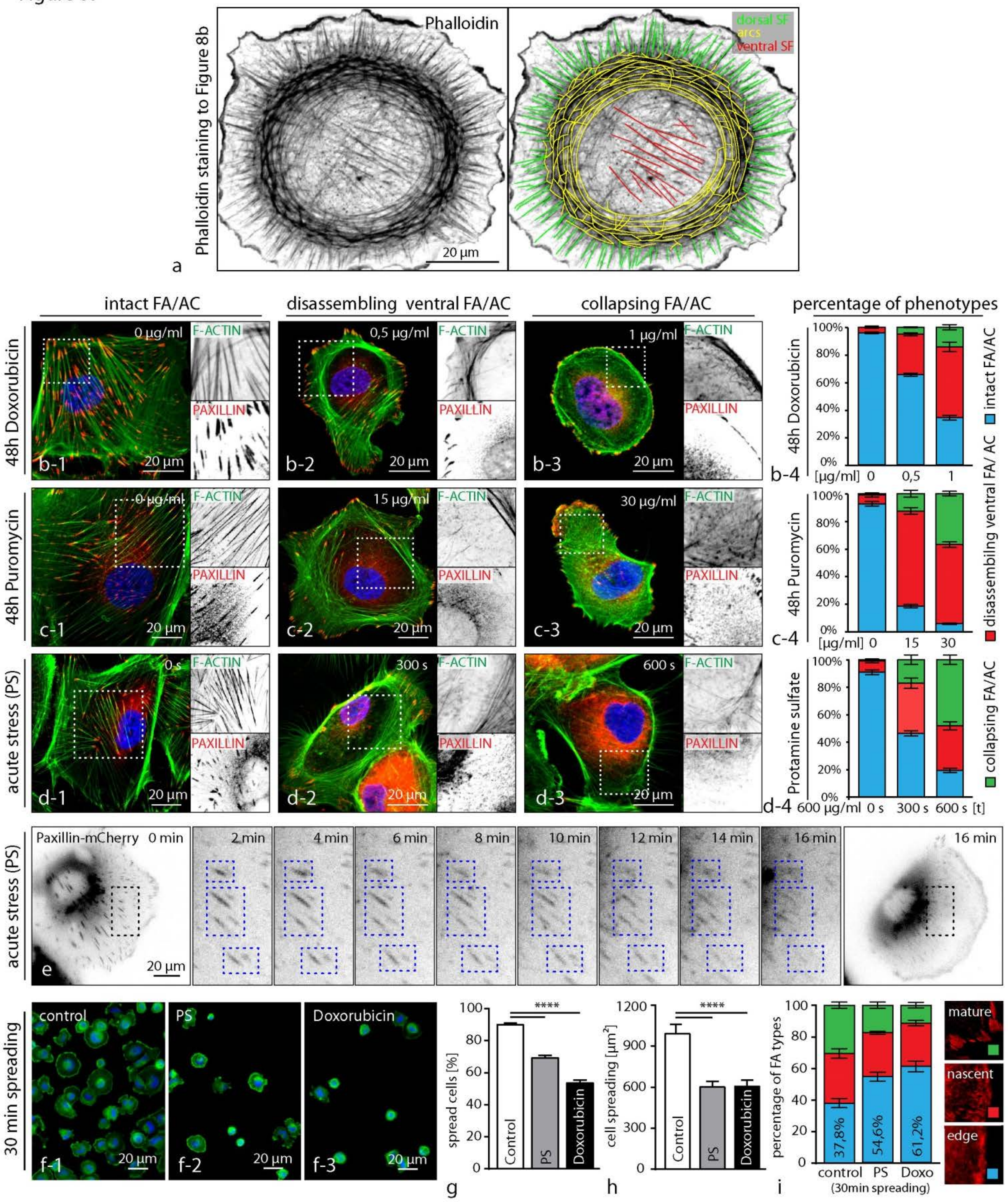
Phalloidin staining to Figure 8b
Phalloidin
dorsal SF
arcs
ventral SF
20 µm
a
intact FA/AC
disassembling ventral FA/AC
collapsing FA/AC
percentage of phenotypes
48h Doxorubicin
0 µg/ml
0,5 µg/ml
1 µg/ml
F-ACTIN
PAXILLIN
b-1
b-2
b-3
b-4
[µg/ml] 0 0,5 1
48h Puromycin
0 µg/ml
15 µg/ml
30 µg/ml
c-1
c-2
c-3
c-4
[µg/ml] 0 15 30
acute stress (PS)
0 s
300 s
600 s
d-1
d-2
d-3
d-4
Protamine sulfate
600 µg/ml 0 s 300 s 600 s [t]
intact FA/AC
disassembling ventral FA/ AC
collapsing FA/AC
Paxillin-mCherry 0 min
2 min
4 min
6 min
8 min
10 min
12 min
14 min
16 min
e
30 min spreading
control
PS
Doxorubicin
f-1
f-2
f-3
spread cells [%]
Control
PS
Doxorubicin
****
g
cell spreading [µm²]
h
percentage of FA types
37,8%
54,6%
61,2%
control PS Doxo
(30min spreading)
mature
nascent
edge
i

Figure S7. The ventral actin cytoskeleton – focal adhesion complex shows high susceptibility to podocyte stress.

(a) Colorized cell indicating the main subsets of fibrillary actin bundles: dorsal stress fibers (SF), arcs and ventral stress fibers. **(b-d)** Application of diverse stress stimuli on immortalized human podocytes resulted in the dissolution of the actin cytoskeleton (AC) and focal adhesions (FA) and reveals a high susceptibility of the ventral actin cytoskeleton and focal adhesions to podocyte stress (at least 100 cells in 3 independent replicates were analyzed, one-way ANOVA with Tukey post-test were performed, results are significant ($p<0.001$) for differences in intact FA/AC group of all 3 treatment experiments). **(e)** Live cell TIRF imaging of PAXILLIN-mCherry expressing human podocyte shows rapid disassembling of focal adhesions after application of 300µg/ml Protamine sulfate. Cells were stained for PAXILLIN and Phalloidin (F-ACTIN). **(f-i)** Cell spreading and focal adhesion maturation of human podocytes was reduced under treatment with Doxorubicin and Protamine sulfate. Cells were stained for PAXILLIN and Phalloidin (F-ACTIN). (At least 100 cells in 3 independent replicates were analyzed for percentage of spread cells, for spread cell area n= 52 control, 53 PS and 51 Doxorubicin cells were analyzed; one-way ANOVA with Tukey post-test were performed, **** $p<0.0001$) Quantification of focal adhesion subtypes revealed that the majority of focal adhesions in doxorubicin treated cells are not fully assembled and showed delayed progression towards maturated adhesions (more than 300 cells per individual condition in 3 independent experiments were evaluated and subtypes were expressed as classes, results are significant for differences of mature focal adhesion group between control and PS treated cells ($p<0.01$) as well as control to Doxorubicin treated cells ($p<0.001$)).

Figure S8

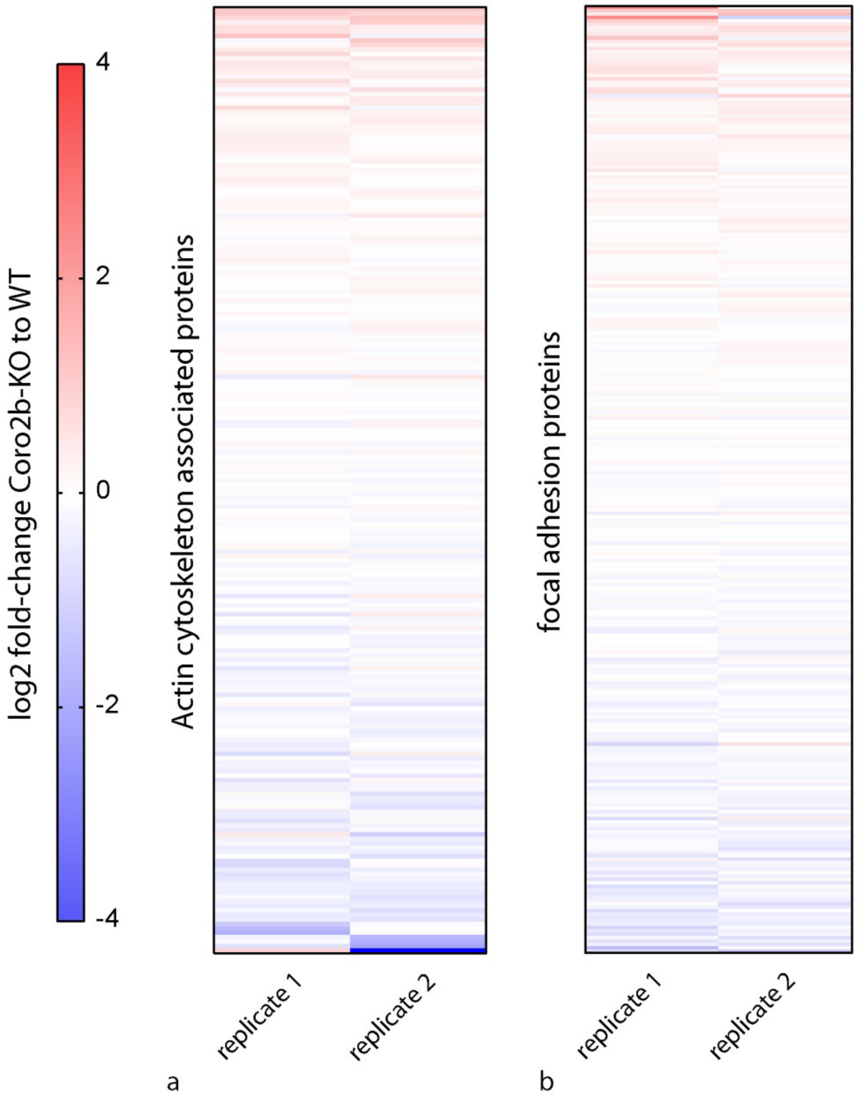

c (consensus focal adhesome)

	replicate 1	replicate 2
Actn1	1.25	-0.32
Rpl23a	0.43	0.31
Limd1	0.42	0.25
Ppib	0.11	0.40
Trip6	0.19	0.31
Hp1bp3	0.13	0.36
P4hb	0.17	0.20
Flnc	0.30	-0.01
Arhgef7	0.43	-0.16
Tns3	-0.11	0.30
Csk	-0.02	0.21
Sorbs3	0.10	0.08
Alyref	0.37	-0.23
Poldip3	0.07	0.02
Pdlim5	-0.02	0.09
Parva	-0.14	0.21
Lims1	-0.17	0.23
Itgav	-0.08	0.14
Ptk2	0.15	-0.09
Tgm2	0.03	0.02
Cnn2	0.09	-0.06
Pxn	-0.04	0.06
Ilk	-0.06	0.07
Tgfb1i1	0.13	-0.12
Vasp	0.19	-0.22
Fhl2	-0.02	-0.07
Fen1	0.02	-0.13
Git2	-0.13	0.01
Syncrip	-0.13	0.001
Pls3	0.05	-0.18
Vcl	0.02	-0.19
Dnajb1	-0.05	-0.13
Cald1	-0.21	-0.02
Itgb3	-0.42	0.19
Zyx	-0.08	-0.18
Dimt1	-0.41	0.14
Actn1	0.02	-0.32
Fermt2	-0.13	-0.19
Fhl3	-0.02	-0.30
Anxa1	-0.18	-0.14
H1fx	-0.62	0.30
Tln1	-0.11	-0.24
Ddx27	-0.29	-0.06
Palld	-0.26	-0.10
Rsu1	-0.33	-0.06
Actn4	-0.24	-0.16
Lasp1	-0.24	-0.18
Pdlim7	-0.10	-0.34
Itgb1	-0.22	-0.22
Iqgap1	-0.11	-0.34
Itga5	-0.17	-0.31
Lpp	-0.17	-0.37
Fblim1	-0.36	-0.25
Brix1	-0.56	-0.14
Tes	-0.46	-0.29
Sorbs1	-0.28	-0.53
Ddx18	-0.51	-0.33
Pdlim1	-0.46	-0.43
Palld	-0.20	-0.79
Cald1	0.07	-2.26

d

	replicate 1	replicate 2
Coro1b	0.17	0.42
Coro1c	-0.22	-0.25
Coro7	0.21	-0.38
Cfl1	-0.11	0.23
Cfl2	-0.25	0.07

Figure S8. Loss of CORO2B does not substantially alter proteome abundance or composition.

(a-d) Whole cell proteomic analysis of *Coro2b* knockout and wildtype MEFs was performed. Concerning focal adhesion or actin cytoskeleton proteins, no alterations of protein abundance or composition was detected. (For detailed information see methods section and supplemental dataset S3).

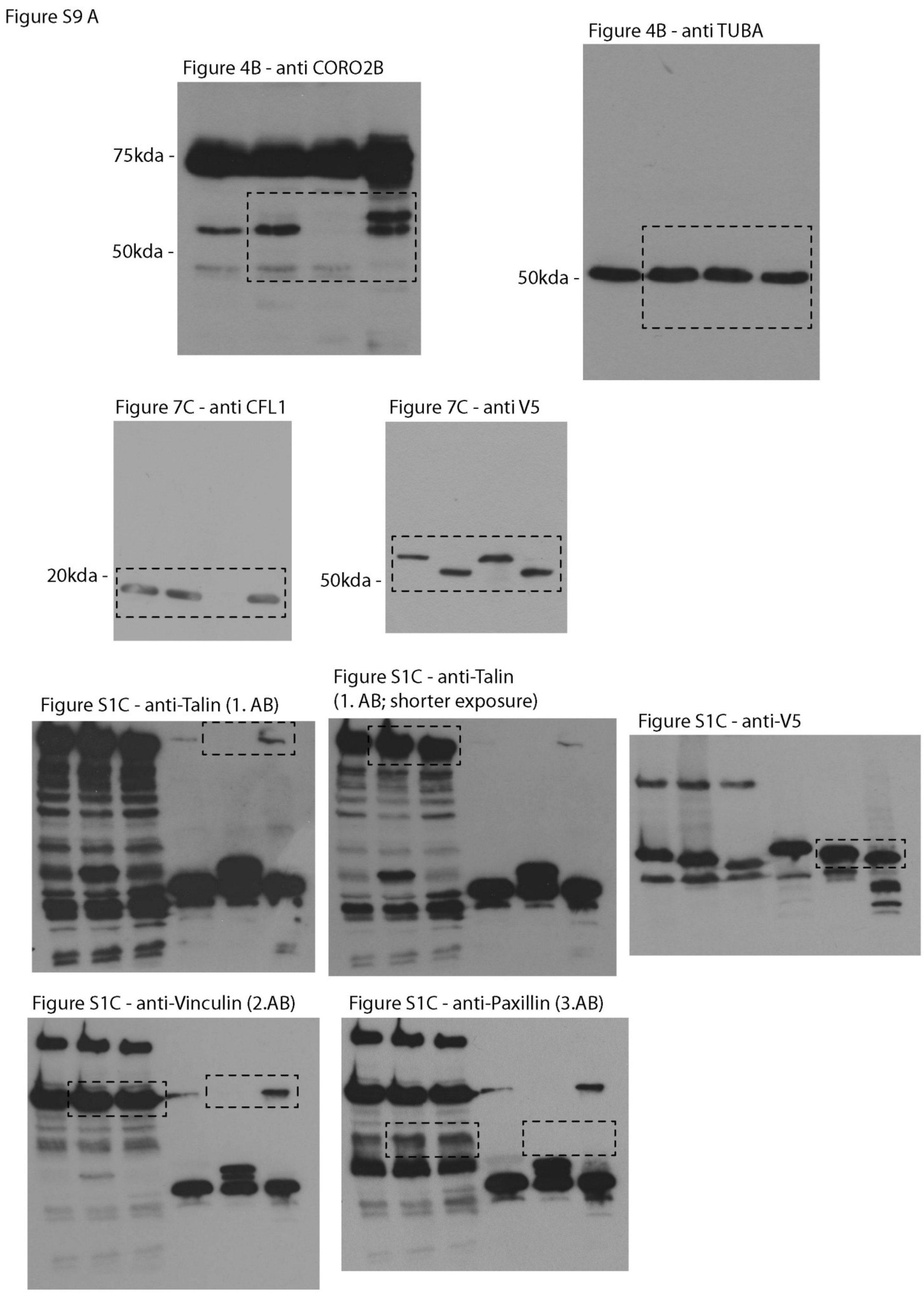

Figure S9 A and B. Uncropped WB gels

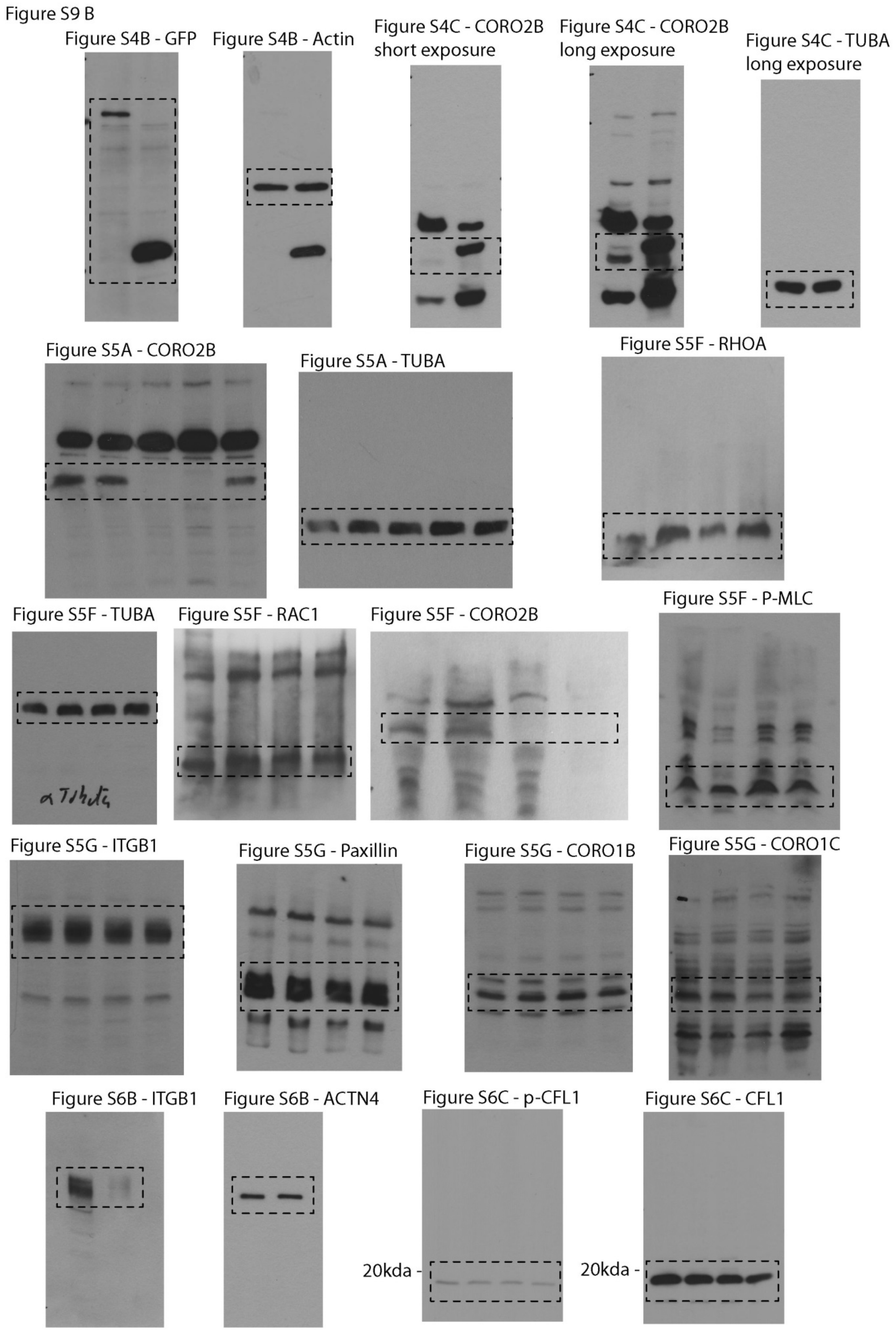

Figure S9 A and B. Uncropped WB gels
